# De novo design of a nanopore for single-molecule detection that incorporates a β-hairpin peptide

**DOI:** 10.1038/s41565-021-01008-w

**Published:** 2021-11-22

**Authors:** Keisuke Shimizu, Batsaikhan Mijiddorj, Masataka Usami, Ikuro Mizoguchi, Shuhei Yoshida, Shiori Akayama, Yoshio Hamada, Akifumi Ohyama, Kenji Usui, Izuru Kawamura, Ryuji Kawano

**Affiliations:** 1 Department of Biotechnology and Life Science, Tokyo University of Agriculture and Technology (TUAT), Tokyo, Japan; 2 Graduate School of Engineering, Yokohama National University, Yokohama, Japan; 3 School of Engineering and Applied Sciences, National University of Mongolia, Ulaanbaatar, Mongolia; 4 Faculty of Frontiers of Innovative Research in Science and Technology (FIRST), Konan University, Kobe, Japan; 5 Graduate School of Engineering Science, Yokohama National University, Yokohama, Japan

**Keywords:** Nanopores, Biomaterials

## Abstract

The amino-acid sequence of a protein encodes information on its three-dimensional structure and specific functionality. De novo design has emerged as a method to manipulate the primary structure for the development of artificial proteins and peptides with desired functionality. This paper describes the de novo design of a pore-forming peptide, named SV28, that has a β-hairpin structure and assembles to form a stable nanopore in a bilayer lipid membrane. This large synthetic nanopore is an entirely artificial device for practical applications. The peptide forms multidispersely sized nanopore structures ranging from 1.7 to 6.3 nm in diameter and can detect DNAs. To form a monodispersely sized nanopore, we redesigned the SV28 by introducing a glycine-kink mutation. The resulting redesigned peptide forms a monodisperse pore with a diameter of 1.7 nm leading to detection of a single polypeptide chain. Such de novo design of a β-hairpin peptide has the potential to create artificial nanopores, which can be size adjusted to a target molecule.

## Abstract (web summary)

De novo designed peptide with β-hairpin structure assembles to form a β-barrelled nanopore that can detect not only polynucleotides but also polypeptide chains at a single-molecule level.

## Main

The folded structure of proteins is determined by their linear polypeptide sequence, as postulated in Anfinsen’s dogma^1^, and gives rise to specific protein functionality. All proteins have a unique structure and size. The folded structure relies on the primary sequence of the amino acids, while this unique primary structure is the result of structural evolution such as the mutation and selection of amino-acid residues over time. To reveal the relationship between this primary information and protein structure is one of the ultimate goals of science.

The de novo design of the primary sequence of artificial proteins has been studied in the last four decades^2–4^, and recently has also been described as design from scratch^5–11^. In early studies, the secondary structures of proteins—α-helix and β-sheet structures—were created synthetically by peptide chemistry^12^, with these secondary structures subsequently connected through a loop sequence to construct the more complicated three-dimensional structure^13^. Although the design strategy at the time was manual and based on the physical model of proteins and peptides, computational design has recently emerged. Baker and coworkers have proposed extensive artificial proteins with computational design, such as a fluorescence-activating protein with a β-barrel^14^ and transmembrane proteins with α-helical^15^ structures. The de novo design has the potential not only to mimic natural proteins, but also to create artificial devices such as molecular machines. In the creation of manufactured devices for practical applications, pore-forming transmembrane structures are meaningful targets because single molecular detection and DNA sequencing have been achieved using such pore-forming proteins and peptides^16–19^.

Nanopore sensing is a powerful tool for label-free single-molecule detection^20,21^. Once a nanopore-forming membrane protein has been reconstituted in a lipid bilayer to form a nano-sized pore, the target molecule is able to electrophoretically pass through the nanopore under an applied voltage. A wide variety of applications have been proposed such as DNA sequencing^22^, small molecule detection using an adapter^23^ or DNA aptamer^24^, nanopore mass spectroscopy^25^, decoding of DNA computations^26–28^ and protein or peptide detection^29,30^. The choice of the applicable target molecule is sometimes limited because the selectivity of nanopore sensing mainly depends on the pore size and chemical properties, and the size variation of natural pore-forming proteins is insufficient for the detection of a range of molecules^31^. Bottom-up nanopore design has great potential to expand target variation, owing to the possibility to tailor size compatibility between the nanopore and target molecules. Moreover, this can offer improved accuracy of nanopore detection, with a potentially substantial influence on amino-acid sequencing of proteins.

In designing an artificial protein nanopore, the process of pore insertion into the lipid membrane must also be considered. In the case of a natural system, membrane proteins are inserted into the cell membrane via chaperones or endoplasmic reticulum export. One way to facilitate membrane insertion is to use short peptides. For example, an α-helical barrelled peptide (35 amino acids) based on the Wza protein has previously been redesigned from the wild type and assembled to form monodisperse nanopores in lipid membranes^18,19^.

Here, we focus on the β-barrel structure of peptides because the transmembrane region of most biological nanopores, including α-hemolysin (αHL), has a β-barrel structure. A transmembrane peptide with a de novo design incorporating a β-hairpin structure was chemically synthesized using an isoacyl dipeptide method^32^. Our designed β-hairpin peptide, named SV28, assembled and formed a β-barrel structure with several different sizes of nanopore ranging from 1.7 to 6.3 nm in diameter. To construct the monodispersely sized pore by designing the amino-acid sequence of the SV28, we introduced a glycine mutation into the strand named SVG28. The SVG28 forms a 1.7 nm pore monodispersely and can detect a single polypeptide. The de novo design of β-barrel nanopores has great potential, with the ability to adjust size and shape to a target molecule, and with applications in the detection of DNA or various other molecules.

## Design of the β-hairpin peptide

**Fig. 1:**
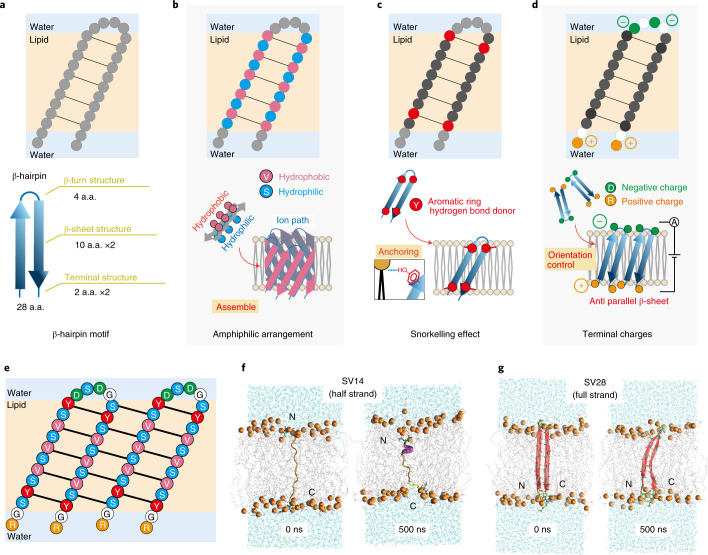
Design strategies and structural confirmation of the β-hairpin peptide. **a**, The design of β-hairpin peptide with 28 amino acids (a.a.) divided into three sections: β-turn, β-sheet transmembrane and the terminals. **b**, Hydrophilic and hydrophobic amino acids are arranged in an alternating fashion. **c**, Interaction of aromatic rings stabilizes the β-barrel pore. **d**, Designing specific charges at the terminus allows control of peptide orientation on an applied voltage. **e**, Amino-acid sequence of the designed structure named SV28. The black lines indicate the hydrogen bonding. **f**,**g**, MD simulations of the monomer structures. The 0 ns (left) and 500 ns (right) snapshots of the half-length (**f**) and full-length (**g**) SV28 in a lipid bilayer membrane.

We designed the β-hairpin structure with three different regions of amino-acid sequence: a β-strand backbone, a β-turn and two terminal structures (Figs. 1a–d). We first considered the length of the peptide. The β-strands are necessary to have appropriate length in the transmembrane region. We decided to use ten amino acids to provide a length compatible with the thickness of a lipid bilayer, because this is similar to the β-strand length of natural transmembrane β-barrel proteins^33^. The β-turn can form a bent structure with four amino acids^34^. The terminal structures require two amino acids with a random coil structure in the extra-membrane regions. Overall, a length of 28 amino acids was selected for use in this study (Fig. 1a). The following three strategies were then used to determine the type of amino-acid residue in each region.

1. Construction of amphiphilic β-sheet structure as the β-strand backbone. Alternation of hydrophilic and hydrophobic amino acids was used to promote formation of the β-sheet structure (Fig. 1b)^35^. Separation of the hydrophilic and hydrophobic surfaces to be inside and outside the nanopore facilitates construction of the β-barrel structure in the lipid bilayer. Ser was selected as the hydrophilic residue because it is the smallest hydrophilic amino acid and may prevent steric hindrance in nanopore formation. Although Ala is the smallest hydrophobic amino acid, it tends to form an α-helical structure and so Val was selected instead as the hydrophobic residue.
2. Stabilization of the membrane-spanning state using the snorkelling effect. In natural α-helix and β-barrel membrane proteins, Trp and Tyr are sometimes localized at the interface between the aqueous and lipid phases, enhancing stability of the transmembrane structure^36^, also known as the snorkelling effect^37^. On the basis of this information, we selected Tyr, with its location carefully chosen at positions 4, 12, 18 and 26 from the N terminus (Fig. 1c).
3. Introduction of charged residues for controlling the orientation of SV28 using an applied voltage. Antiparallel β-sheets have more strong interactions than parallel β-sheets. To control the peptide orientation for forming the antiparallel β-sheets structure, two negatively and positively charged residues were introduced at the β-turn and the terminal regions, respectively (Fig. 1d). The β-turn consists of four amino acids and the systematic study of the β-turn sequence has been previously reported^34^, so we decided on the sequence of –DSDG–. The N- and C-terminal regions were designed as RG– and –GR, respectively. Gly is the linker between charged Arg residues connecting to the β-sheet backbone.

The final sequence of SV28 is shown in Fig. 1e. The β-hairpin formation of the SV28 sequence was computationally confirmed using the MINNOU simulator, software that predicts transmembrane domains of membrane proteins and peptides^38^.

**Fig. 2:**
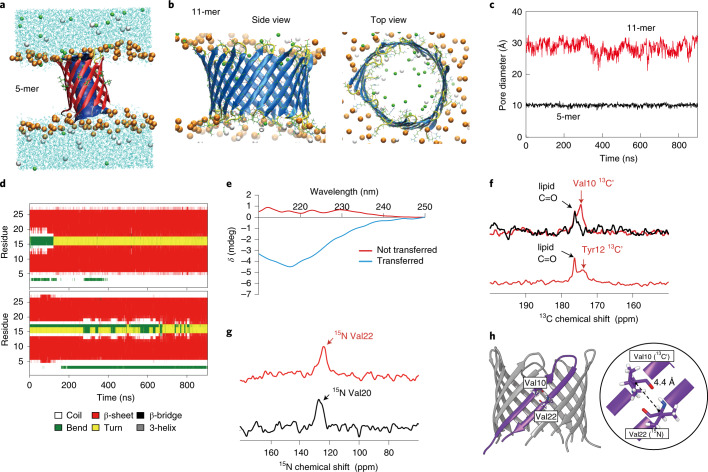
Prediction and confirmation of the assembling structure of the SV28 peptides in the lipid bilayer membrane. **a**, The last snapshot of 5-mer SV28 in a DOPC membrane in the MD simulation. Ribbons show the peptide structures, with the secondary structure indicated by the colour of the ribbon (red, β-sheet; yellow, turn and white, random coil structure). Ribbon arrows indicate the direction of the backbone from N to C terminal. Cyan lines indicate water molecules, and the lipid molecules were omitted for clarity (excluding phosphorus atoms as orange spheres). Green and white spheres indicate the potassium and chloride ions, respectively. **b**, MD simulation of the SV28 nanopore formation in the DOPC lipid membrane. The 11-mer nanopore was simulated for 900 ns after 100 ns of initial simulation. Brown, lipid head; green, potassium ions and grey, chloride ion. **c**, Central diameters of 5-mer (black) and 11-mer (red) pores as a function of time were calculated using HOLE software at 1 ns intervals. **d**, Profile of the secondary structure of 5- and 11-mer pores as a function of time. **e**, Circular dichroism spectra of non-transformed SV28 (red line) and transformed SV28 (blue line). **f**, ^1^^3^C CP-MAS NMR spectra of the triply isotope-labelled SV28 ([1-^13^C]Val10, [2-^13^C]Gly16, [^15^N]Val22-labelled and [1-^13^C]Tyr12, [2-^13^C]Gly16, [^15^N]Val20-labelled SV28) in DOPC liposomes. The black lines indicate the spectra from DOPC liposomes, and the red lines indicate the spectra of isotope-labelled SV28 with DOPC liposomes. **g**, ^15^N CP-MAS NMR signals of Val20 and Val22 of SV28. **h**, The β-barrel structure of SV28 nanopore. The interatomic distance between the backbone amide nitrogen of Val22 and the carbonyl carbon of Val10 in the SV28 is estimated to be around 4.4 Å as measured by solid-state NMR.

We verified the formation of the nanopore structure from assembled half- and full-length of SV28 to confirm the stability of the β-hairpin structure in the membrane (Fig. 1f,g) by all-atom molecular dynamics (MD) simulations. The full-length SV28 monomer kept the β-hairpin structure, while the half-length mostly showed random coil secondary structures during these simulations (Supplementary Fig. 1). To confirm the β-barrel formation of SV28 peptides, we performed 1,000 ns MD simulations of 5- and 11-mer pores in a dioleoylphosphatidylcholine (DOPC) membrane. Figure 2a and Supplementary Fig 2 display the final snapshots of these simulations, with the 11-mer structure also shown in Fig. 2b. Both simulations demonstrated stable nanopore structures. The 5-mer pore was slightly tilted in the membrane in Fig. 2a, which may indicate the adaptation of the structure to the movement of surrounding lipids. The central diameter of the pores was calculated at 1 ns intervals, as shown in Fig. 2c and Supplementary Fig 2. The average values are equal to 10.1 ± 0.4 and 28.5 ± 2.0 Å for 5- and 11-mer pores, respectively.

Furthermore, the secondary structure profiles of two monomers of the pores as a function of time are shown in Fig. 2d. The tenth (Val10) and 22nd (Val22) positions form a β-sheet structure, and the 16th position (Gly16) forms a turn structure while the turn sequence seems to be slightly shifted. To compare with the experimental results, we calculated the distance between the nitrogen of Val10 and carbon of Val22 during the simulations (Supplementary Fig. 3a). The average distances of the 5- and 11-mer peptides are 4.2 ± 0.1 and 4.1 ± 0.1 Å, respectively. These distances are consistent with the usual value of the distance in the β barrels of outer membrane proteins. MD simulations of other oligomerized pores, 7- and 16-mer, are also shown to give stable pore formation as shown in Supplementary Fig. 3b,c.

## Synthesis of a hydrophobic β-hairpin peptides using isoacyl dipeptide

Conventional solid-phase synthesis and subsequent purification of SV28 is challenging because the β-sheet structure is prone to aggregation. A method using isoacyl dipeptides^32^ gives the aqueous-soluble SV28 preform; the final chemical structure can be derived from this preform after transition from the side to the main chain in the isoacyl peptide (Supplementary Fig. 4). In this way, it was possible to synthesize and purify SV28 using the conventional peptide synthesis method (Supplementary Fig. 5). The ester bond was transferred to a peptide bond by incubation in a basic solution (pH 13) for 5 min before using the peptides in the experiments. Acyl migration was confirmed using reversed-phase high-performance liquid chromatography (Supplementary Fig. 6).

The secondary structure of the transferred SV28 precursors with DOPC liposomes was confirmed by circular dichroism spectroscopy under similar conditions to the following channel current measurements. The SV28 preform did not show any large negative Cotton effect at 216 nm (Fig. 2e and Supplementary Fig 7). After the transference of isoacyl dipeptide, a large negative Cotton effect at 216 nm was observed (Fig. 2e and Supplementary Fig 7), indicating that the transformation of isoacyl dipeptide facilitated the formation of a β-sheet structure in SV28. Moreover, after 24 h incubation in a buffer solution, the peptides were seen to maintain the β-sheet structure (Supplementary Fig. 7).

The solid-state nuclear magnetic resonance (NMR) measurements also supported the proposed conformation of SV28 in DOPC liposomes. ^13^C and ^15^N NMR signals of [^13^C]Val10, [^13^C]Gly16 (Fig. 2f and Supplementary Fig 8), and [^15^N]Val22 were observed at 174.2, 45.3 and 124.1 ppm (Fig. 2g), corresponding to the β sheet and the random coil structure at the transmembrane (Val10 and Val22) and the β-turn (Gly16) region^39^. Next, we confirmed the formation of hydrogen bonding between two β strands using Val10 and Val22 (Fig. 2h), with the distance between them also checked by MD simulation as mentioned above. Rotational echo-double resonance was used to estimate the interatomic distance between the carbonyl carbon of Val10 and amide nitrogen of Val22 in SV28 to be around 4.4 Å (Supplementary Fig. 9), indicating the formation of hydrogen bonds between the β strands.

The NMR-deduced structure approximately corresponds to the structure predicted by the MD simulation. Overall, the information indicates the formation of the β-hairpin structure of SV28.

## Confirmation of pore formation using channel current measurements

**Fig. 3:**
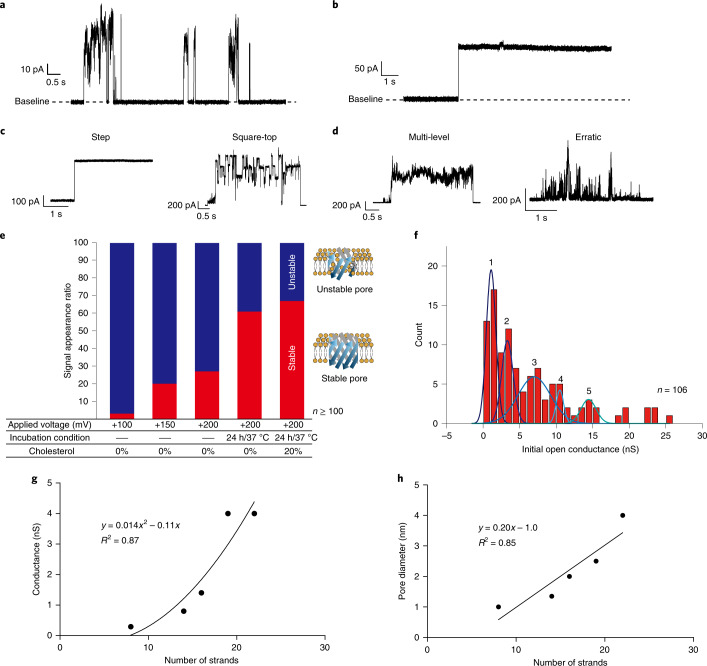
Evaluation of the stability and conductance of the SV28 nanopores using channel current measurements. **a**, The typical current and time traces of SV28 at +100 mV with initial condition. **b**, The step signal was occasionally observed under the initial conditions. The traces were atypical before optimization of the conditions. **c**, Step and square-top signals were defined as stable pore formation. **d**, Multi-level and erratic signals were defined as unstable pore formation. **e**, The ratio of stable and unstable pore formation from the channel current analysis. **f**, The histogram of the current conductance of the initial step from the 0 A increase of SV28 in the DOPC lipid bilayer. **g**,**h**, The relationships in β-barrel membrane proteins OmpA, OmpG, OmpF, VDAC and FhuA between the channel conductance (**g**) and the number of β strands and the pore diameter and number of β strands (**h**).

The pore-forming properties of SV28 were examined by the channel current recording in our lipid bilayer system (Supplementary Fig. 10)^40^. Several pore-opening states in which the current was raised, but did not plateau, were observed in the DOPC lipid bilayer under +100 mV (Fig. 3a). Sometimes step-like signals were observed (Fig. 3b). Other different shapes of current signals were also observed. We have previously proposed current signal classification for several different signal shapes, and assigned these to various pore-forming models of α-helical peptides^41–43^. In this study, we also classified these signals into four types of current signal: step, square-top (Fig. 3c), multi-level and erratic (Fig. 3d). The definition of signal classification is described in Supplementary Fig. 11. To estimate the pore-forming behaviour of SV28, we here define that step and square-top signals reflect stable pore formations, and multi-level and erratic signals reflect unstable pore formations. In the initial measurement of SV28, the stable signals were observed only 3% of the time (Fig. 3e, left bar), which led us to investigate how to improve stable pore formation. To optimize formation of stable pores, we investigated three different conditions as follows (Fig. 3e and details in Methods):

1. Optimization of applied voltage to align the SV28 monomers.
2. Incubation of SV28 monomers to form oligomer structures with the lipid monolayer surface.
3. Adding cholesterol to the lipid bilayer.

Combining the three optimized conditions led to a further increase in the ratio of stable signals to 67% (Fig. 3e, right). This ratio is close to the ratio we previously reported for pore-forming proteins^31^. The other typical current signals and step signals are shown in Supplementary Figs. 12 and 13.

The pore diameter of the SV28 nanopore was calculated using the conductance of the open channel state. The open channel conductance was determined as the initial step signal from the baseline (≅0 A). The histogram of the pore conductance of SV28 is shown in Fig. 3f. Several peaks are observed in this histogram, with five identified peaks picked by the second deviation method. The peak conductances at 1, 3, 7, 11 and 14 nS are identified. We estimated the pore size using the experimental results instead of the theoretical Hille model. We assessed the relationship between the current conductance and the pore diameter of β-barrel proteins, measurements that were taken by electrophysiology and by crystallography using microscopes. OmpA, OmpF, OmpG, VDAC and FhuA were used in this estimation as the β-barrel transmembrane proteins. On the basis of the relationship between the current conductance and the pore diameter of the natural proteins (Fig. 3g,h), the five different pore sizes and the number of monomers in the case of SV28 are approximately estimated as 1.7, 3.0, 4.4, 5.4 and 6.3 nm with 7, 10, 13, 16 and 18 monomers of SV28, respectively (Table 1). The experimentally estimated pore sizes are larger than those from the theoretical Hille model.

**Table 1:** Table of conductance values showing the number of monomers, strands and the pore diameters of the SV28 nanopores

Peak no.	Conductance (nS)	Monomer no.	Strand no.	Diameter (nm)
1	1.1	6.9	13.8	1.7
2	3.3	9.9	19.8	3.0
3	7.0	13.3	26.6	4.4
4	10.5	15.8	31.6	5.4
5	14.4	18.2	36.4	6.3

## Detection of double-stranded DNA and G4 structure of DNA using the SV28 nanopore

**Fig. 4:**
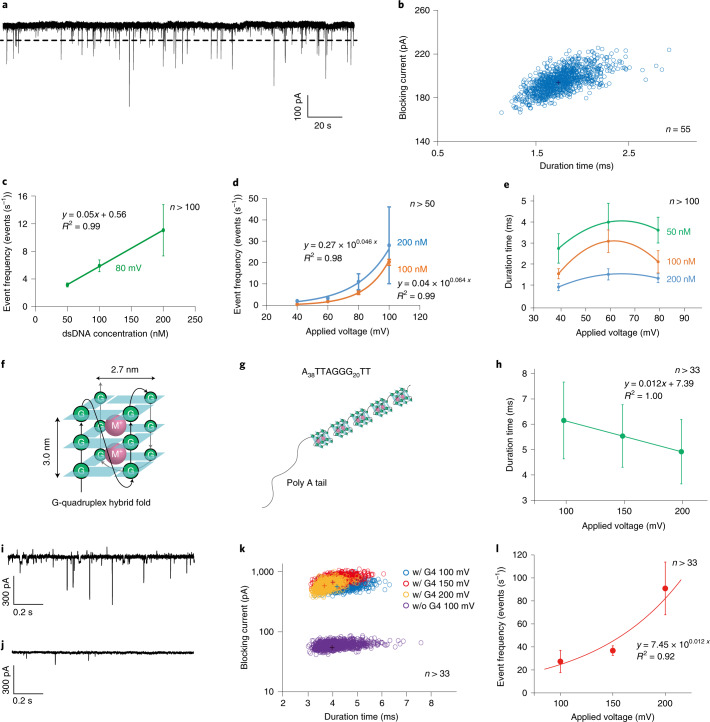
The dsDNA (1 kbp) translocation through the SV28 nanopore with diameter of around 5 nm. **a**, The current and time trace of the SV28 nanopore with 1 kbp dsDNA (100 nM) under application of 40 mV. The dashed lines indicate the threshold for the dsDNA translocation events. **b**, The scatter plot of the translocation data of 100 nM and 100 mV after bootstrapping. **c**, The event frequency of the translocation as a function of the concentration of dsDNA. **d**, The event frequency of the dsDNA translocation as a function of the applied voltage. Blue and orange lines indicate the dsDNA concentration at 100 and 200 nM to guide the eye. **e**, Duration time of dsDNA with 50, 100 and 200 nM dependence on the applying voltages. Blue, orange and green lines indicate each concentration to guide the eye. **f**,**g**, Schematic structure of G4 and (**f**) five G4s (**g**) in the hybrid hold in a series. **h**, Duration time of the G4 dependence on the applying voltages at 100, 150 and 200 mV. **i**,**j**, The current and time trace of the SV28 nanopore with (**i**) and without (**j**) G4. **k**, Scatter plots of the blocking current and duration time after bootstrapping of DNA with (w/) G4 and without (w/o) G4 structure. The peak tops of each condition with G4 are (duration, blocking current of 4.24 ms, 669 pA) at 100 mV (4.07 ms, 797 pA), 150 mV, (3.72 ms, 678 pA) 200 mV and without G4 (4.05 ms, 62 pA) at 100 mV. **f**, The duration time of G4 (2 μM) as a function of the applying voltages at 100, 150 and 200 mV. **l**, The event frequency of the translocation of G4 (2 μM) as a function of the applied voltages. The lines in **c**–**h**,**l** show the results of fitting by liner or exponential models.

We used SV28 with a pore diameter of around 5.4 nm and attempted to detect double-stranded DNA (dsDNA) with lengths of 50 basepairs (bp) and 1 kilobasepairs (kbp). Although the translocation signal was not discernible by the inherent blockage of the pore in the case of 50-bp dsDNA measurements at 1 and 10 μM concentrations (Supplementary Fig. 14), the blocking currents were consistently observed using 1-kbp dsDNA ranging from 50 to 200 nM (Fig. 4a and Supplementary Fig 15). The scatter plot of the blocking rate and duration time of translocation at 100 nM under application of 100 mV is shown in Fig. 4b, with peak values of 195 pA and 1.8 ms, respectively (scatter plots without bootstrapping are presented in Supplementary Fig. 16). Additionally, the translocation of multiple dsDNA through the pore was occasionally observed (Supplementary Fig. 17). We next examined the dependency of the translocation frequency of dsDNA on the concentration and applied voltage. The event frequency versus concentration showed a linear dependency ranging from 50 to 200 nM under the application of 80 mV (Fig. 4c). Moreover, the event also depended exponentially on the voltage application from 40 to 100 mV at concentrations of both 100 and 200 nM (Fig. 4d). These linear and exponential dependencies on the concentration and applied voltage are consistent with previously reported results of DNA translocation using the αHL nanopore^44^. The voltage dependency of the duration time did not show a simple decrease with voltage application, but had a peak voltage (Fig. 4e). A similar result has previously been reported, and may be due to rejection of the target molecule from the pore under low voltage application^45^. The scatter plots from all data are shown in Supplementary Fig. 18.

Since the SV28 forms several different sizes of nanopore, here we detect the G4 structure of DNA using a large SV28 nanopore (6.4 nm). The G4 DNA forms a hybrid hold with a 3.0 × 2.7 nm^2^ box-like structure and the G4 structure is strung out as five boxes in this salt condition (Fig. 4f,g)^46,47^. In the case of the G4 structure, the blocking signals are observed, although the same length of DNA without G4 structure is not recognized as shown in Fig. 4i,j. The scatter plots with and without the G4 structure are markedly different (Fig. 4k). The peak top of the blockade of the G4 structure is 669 pA at 100 mV of applied voltage. The ratio of the blocking amplitudes between dsDNA and G4 is *I*_dsDNA_/*I*_G4_ = 0.3 (195/669 pA). This value roughly consists of the ratio of their molecular volume: *V*_dsDNA_/*V*_G4_ = 0.4 (12.5 nm^3^/29.2 nm^3^ and 4 nm long). The dwell time and translocation frequency of G4 depend on the applied voltages (Fig. 4h,l). The area of the scatter plots at different voltages is seen to occur at in a different location in the graph in Fig. 4k. These scatter areas are also located at different positions to the inherent current blocking by the SV28 nanopore (Supplementary Fig. 19). The SV28 nanopore can discriminate the human telomeric DNA at the single-molecule level.

## Re-design of SV28 with a glycine-kink and poly-lysine detection using the SVG28 nanopore

**Fig. 5:**
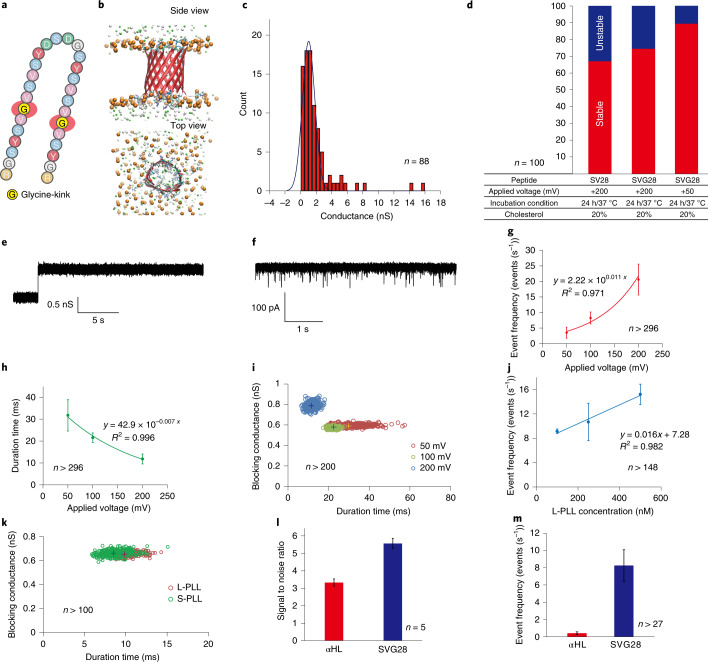
The SVG28 structure and sensing capability for a poly-arginine. **a**, Sequence of the SVG28 that has G-kink into the transmembrane region. **b**, The structure of the SVG28 in a DOPC membrane in the MD simulation from side and top views. **c**, The histogram of the current conductance of the initial step from the baseline level (0 A) increase of SVG28 in the DOPC lipid bilayer. **d**, The ratio of stable and unstable pore formation from the channel current analysis with comparing to the SV28 and the SVG28. **e**,**f**, Typical current and time trace of the SVG28 nanopore with L-PLL (**f**) and without L-PLL (**e**). **g**, The event frequency of the L-PLL detection as a function of the applied voltages. **h**, Duration time of the L-PLL detection dependence on the applying voltages at 50, 100 and 200 mV. **i**, The scatter plot of the L-PLL detection in 100 nM: the blocking rate and the duration after the bootstrapping. **j**, The event frequency as a function of the concentration of the L-PLL. **k**, The scatter plot of the L-PLL and shorter length of poly-l-lysine (molecular weight roughly 10,000) detection in 250 nM: the blocking rate and the duration after the bootstrapping. **l**, Comparison of the signal to noise ratio on the L-PLL detection between αHL and SVG28 nanopore. **m**, Comparison of the event frequency on the L-PLL detection between αHL and SVG28 nanopores.

Although the SV28 nanopore forms five different-sized pores and can detect differently sized target molecules, the construction of a monodisperse sized pore is a significant challenge in the de novo design. To realize monodisperse pore formation, we redesigned the β-hairpin peptide by introducing a Gly-kink into the transmembrane region of the SV28, named SVG28 (Fig. 5a and Supplementary Fig 4b). A Gly-kink is an intrinsic residue in both natural and de novo proteins with a β-barrel structure^14,48^ that increases the local β-sheet curvature, resulting in stabilization of the β-barrel conformation^14^. We initially confirmed the pore structure of the SVG28 using MD simulation (Fig. 5b and Supplementary Fig 20) and synthesized it using the isoacyl dipeptide method (Supplemental Information and Supplementary Figs. 4f, 5d and 6c). After the transference of isoacyl dipeptide, a large negative Cotton effects at 216 nm was also observed in circular dichroism (Supplementary Fig. 7c), indicating the formation of a β-sheet structure in SVG28.

In the channel current measurements, the SVG28 showed a step signal with a single-channel conductance (Fig. 5c,e and Supplementary Fig 21), indicating more monodisperse pore formation while large pores were rarely observed at the lower voltage application. The main channel conductance of the SVG28 pore (*g* = 1.0 nS) almost corresponds to the smallest pore of the SV28 (peak no. 1 *g* = 1.1 nS, pore diameter, 1.7 nm in Fig. 3f and Supplementary Fig 21f), and the size of the pore can be approximately estimated as 1.7 nm with the same assessment using natural protein pores that are predicted to form a 7-mer pore. Moreover, the ratio of the stable pore formation of SVG28 was higher than that of the SV28 nanopore (Fig. 5d); the ratio reached around 90%, which is almost the same as the alpha-hemolysin (αHL) nanopore^31^. The results support the hypothesis that a G-kink makes a flexure in the β strand and stabilizes the β-barrel structure. In addition, the size of the 7-mer SVG28 nanopore was estimated to be around 1.3 nm using MD simulations (Supplementary Fig. 20).

We next attempted to detect a polypeptide chain using the size-compatible SVG28 nanopore, because the next main target of nanopore sensing is in the amino-acid sequencing of proteins^49^. The translocation-like blocking events of a poly-l-lysine (L-PLL, molecular weight 30,000–70,000) at 100 nM were observed under an applied voltage of 100 mV (Fig. 5f and Supplementary Fig 22). The blocking event frequency (Fig. 5g) and the duration time (Fig. 5h) depended on the applied voltage as well as the SV28 nanopore measurements. The scatter plots of the current blocking and duration also show the voltage dependency of the L-PLL measurement (Fig. 5i), and the event frequency also depended on the concentration (Fig. 5j). These results indicate that the SVG28 nanopore can detect L-PLL as a nanopore sensor. Furthermore, the shorter length of poly-l-lysine (molecular weight roughly 10,000) can also be detected using the SVG28 nanopore (Fig. 5k and Supplementary Fig 22h). Next, we assessed the capability of polypeptide detection compared with the αHL nanopore, which is the most popular nanopore protein for single-molecule detection. Figure 5l,m presents the signal to noise ratio of the blocking events and the event frequency in the L-PLL measurement. Both detection parameters of SVG28 are much better than that of the αHL nanopore for polypeptide detection. Specifically, the event frequency in the SVG28 pore was high, probably due to the electrostatic effect between the L-PLL and the entrance of the nanopore. The αHL has positive charges at the entrance of the pore, which inhibit the entrance of polycations. In contrast, the SVG28 has a negative charge at the entrance, resulting in a high event frequency.

## Conclusions

We designed a pore-forming peptide with a β-sheet structure that forms five differently sized or monodispersely sized nanopores in the lipid membrane. A particularly pleasing result was that we achieved success with our initially designed sequence of SV28; we did not need to adjust this initial sequence via trial-and-error experiments, and we did not need to design many different sequences of peptides as we had initially anticipated. We consider the reason that the reduction of the information hierarchy is one of the keys to our design; in other words, the key is a reduction of the amino-acid sequence space. Our nanopore structure was designed with several limitations and requirements: a β-sheet structure, amphiphilic properties and strong interaction between monomers. Moreover, the length of our peptide was decided as less than 30 amino acids so as to be suitable for chemical synthesis. These requirements limited the sequence and variety of amino acids incorporated into the transmembrane nanopore design.

Design of the peptide nanopore provides insight into the application of nanopore sensing of DNA secondary structures and polypeptides, and also of hurdles encountered in nanopore technology. Our design strategy could also be integrated in the creation of molecular machines with use as part of a molecular robot^50^.

## Methods

### MD simulation

The MD simulations of β-barrel structures of SV28 and SVG28 were performed in a DOPC membrane using GROMACS-5.1.4 and GROMACS-2021.1 (ref. ^51^) and Charmm36 force field^52^. The structural modelling and simulation details are included in the Supplementary Information.

### Preparation of the bilayer lipid membrane (BLM) and peptide pretreatments

BLMs were prepared by the droplet contact method using a microdevice^53,54^. First, the DOPC (lipids/*n*-decane, 10 mg ml^−1^) solution (2.3 μl) was poured into each chamber. Next, the buffer solution (4.7 μl) without any peptide was poured into the recording chamber. The buffer solution (4.7 μl) with SV28 and SVG28 (final concentration 1 μM) was poured into the ground chamber. In this study, a buffer solution (1 M KCl, 10 mM MOPS, pH 7.0) was used. Before the measurement, the SV28 and SVG28 were added to 100 mM KOH and incubated for 5 min to allow transfer of isoacyl dipeptide to the native dipeptide of Val and Ser. Then, HCl was added to make the buffer pH 7. A few minutes after adding the buffer solution, the two lipid monolayers connected to form BLMs. When the BLMs ruptured, they were reconstituted as BLMs by tracing with a hydrophobic stick between the two droplets. The solutions were prepared comprising 2 μM transformed SV28 and SVG28, 1 M KCl, 10 mM MOPS and 10 mg ml^−1^ DOPC or DOPC:cholesterol, 4:1 (w/w) in *n*-decane at pH 7. The solution was agitated in a vortex for 30 s and incubated for 24 h at 37 °C. The lipid and buffer solutions were added to the ground chamber. The details are included in the Supplementary Information.

### Channel current measurements and data analysis

The channel current was monitored using a JET patch-clamp and PICO2 amplifier (Tecella) connected to the chambers. Ag/AgCl electrodes were already present in droplets when the solution was added to the chambers^55^. A constant voltage ranging from 40 to 200 mV was applied to the recording chamber, and the other chamber was grounded. Pore formation in BLMs allowed ions to pass through the nanopore under the voltage gradient, giving the channel current signal. The signals were detected using a 4-kHz low-pass filter at a sampling frequency of 20 kHz. Analysis of channel current signals and duration time was performed using pCLAMP v.11.0.3 (Molecular Devices), Excel (Microsoft) software and in-house programs coded using Python. Channel current measurements were conducted at 22 ± 2 °C. Peaks of conductance histograms were identified by the second derivative method (threshold 15%) and fitted by nonlinear and Gaussian curve fitting methods using OriginPro8.5j (Light Stone). The scatter plots of the blocking current and duration of the DNA translocation and PLL translocation are described after bootstrapping^26^. The bootstrap method is based on the resampling of the original random sample drawn from a population with an unknown distribution. We used the exact bootstrap method, which used the entire space of resamples. In the exact bootstrap method, the accuracy is verified with a sample number over 30. In this study, our bootstrap procedure took the same amount of data randomly from the translocation data, and the mean values for these samples were calculated 65,536 times. The details are included in the Supplementary Information.

### The optimization of the SV28 pore formation

1. Optimization of applied voltage to align the SV28 monomers. It is proposed that the orientation of SV28 can be controlled by applying a voltage, since we designed the positively and negatively charged amino acids to be positioned at the turn and terminal regions. As predicted, the ratio of stable signals increased with increasing the applied voltage from +100 to +200 mV (Fig. 3e).
2. Incubation of SV28 monomers to form oligomer structures with the lipid monolayer surface. To give an energetically stable structure of the assembled SV28, we attempted to incubate the SV28 monomers in a mixed ‘aqueous’-‘lipid/*n*-decane’ solution. We expected the SV28 monomers to assemble at the interface between aqueous and lipid/*n*-decane solution and form a stable pore during incubation. After incubating for 24 h at 37 °C, the ratio of the stable signals dramatically increased from 27 to 61% (Fig. 3e). A similar phenomenon was previously reported wherein amyloid β(1–42), channel-forming β-sheet peptide, forms stable pores after incubation with lipid micelles^56^.
3. Adding cholesterol to the lipid bilayer. Cholesterol addition enhanced the formation of stable pores (Fig. 3e). This result may be explained in that cholesterol addition results in reduced fluidity of the DOPC bilayer, and subsequently there is reduced disturbance and pore dissociation.

## Online content

Any methods, additional references, Nature Research reporting summaries, source data, extended data, supplementary information, acknowledgements, peer review information; details of author contributions and competing interests; and statements of data and code availability are available at 10.1038/s41565-021-01008-w.

## Supplementary information

Supplementary Information Supplementary Text, Figs. 1–22 and References.

## Data Availability

The data that support the plots in this paper and other findings of this study are available from the corresponding author on reasonable request.
