# Supplementary material for: De novo design of a nanopore for single-molecule detection that incorporates a β-hairpin peptide
Source: Nat Nanotechnol. 2021 Nov 22;17(1):67–75. doi: 10.1038/s41565-021-01008-w (PMC8770118; doi:10.1038/s41565-021-01008-w)
Supplement: Supplementary file 1 — Supplementary Text, Figs. 1–22 and References. [file 41565_2021_1008_MOESM1_ESM.pdf]

nature portfolio

https://doi.org/10.1038/s41565-021-01008-w

Supplementary information

De novo design of a nanopore for single-molecule detection that incorporates a β-hairpin peptide

In the format provided by the authors and unedited

Supplementary information

De Novo Design of a Nanopore for Single-Molecule Detection that Incorporates a β-Hairpin Peptide

Keisuke Shimizu[1], Batsaikhan Mijiddorj[2,3], Masataka Usami[1], Ikuro Mizoguchi[1], Shuhei Yoshida[4], Shiori Akayama[4], Yoshio Hamada[4], Akifumi Ohyama[5], Kenji Usui[4], Izuru Kawamura[2,5], and Ryuji Kawano[1*]

[1]*Department of Biotechnology and Life Science, Tokyo University of Agriculture and Technology, Japan*
[2]*Graduate School of Engineering, Yokohama National University, Japan*
[3]*School of Engineering and Applied Sciences, National University of Mongolia, Mongolia*
[4]*Faculty of Frontiers of Innovative Research in Science and Technology, Konan University, Japan*
[5]*Graduate School of Engineering Science, Yokohama National University, Japan*

1. Materials and Methods
   1.1 Reagents and chemicals
   1.2 MD simulations (**Fig. S1**)
   1.3 Secondary structure of SV28 in lipid bilayer membrane (**Fig. S2 and S3**)
      1.3.1 Pore state of 11-mer SV28 in lipid bilayer membrane
      1.3.2 Intrapeptide distance between Val10 and Val22 in 5-mer and 11-mer
      1.3.3 Other assembled SV28 pore: 7-mer and 16-mer (**Fig. S3**)
   1.4 Synthesis of Fmoc-1-^{13}C Tyr(tBu)-OH, Fmoc-2-^{13}C Gly-OH, and Fmoc-1-^{13}C Val-OH
      1.4.1 Fmoc-1-^{13}C Tyrosine
      1.4.2 Fmoc-1-^{13}C Tyr-OCH_2CCl_3
      1.4.3 Fmoc-1-^{13}C Tyr(tBu)-OCH_2CCl_3
      1.4.4 Fmoc-1-^{13}C Tyr(tBu)-OH
      1.4.5 Fmoc-2-^{13}C Gly-OH and Fmoc-1-^{13}C Val-OH
   1.5 Solid-phase synthesis of *O*-isoacyl SV28 and SVG28 precursors (**Fig. S4 and S5**)
   1.6 Confirmation of acyl migration by HPLC (**Fig. S6**)
   1.7 Liposome preparation for CD measurements
   1.8 CD spectroscopy (**Fig. S7**)
   1.9 Solid-state NMR measurements (**Fig. S8 and S9**)
      1.9.1 Confirmation of secondary structure of β-turn
      1.9.2 REDOR for measuring distance between Val10 and Val22
   1.10 Fabrication of microdevices of lipid bilayer system (**Fig. S10**)
   1.11 Preparation of bilayer lipid membrane and alpha-hemolysin pretreatments
2. Channel current measurement (**Fig. S11**)
   2.1 Preparation of dsDNA with 1 kbp and ssDNA with and without G4 DNA
   2.2 Preparation of bilayer lipid membrane and SVG28 pretreatments
   2.3 Detection of poly-L-lysine
3. Single-molecule detection using SV28 and SVG28 nanopore (**Fig. S12 – S19**)
4. MD simulation of SVG28 nanopore (**Fig. S20**)
5. Detection of poly-L-lysine using SVG28 nanopore (**Fig. S21 and S22**)
6. References

1.1 Reagents and chemicals

The following reagents were used: 1,2-dioleoyl-*sn*-glycero-3-phosphocholine (DOPC, Avanti Polar Lipids, USA); 1,2-diphytanoyl-*sn*-glycero-3-phosphocholine (DPhPC, Avanti Polar Lipids, USA); cholesterol (Sigma-Aldrich, USA); *n*-decane (FUJIFILM Wako Pure Chemical Industries, Ltd. (Wako), Japan); dimethyl sulfoxide (DMSO, FUJIFILM Wako Pure Chemical Industries, Ltd. (Wako), Japan) 3-morpholinopropane-1-sulfonic acid (MOPS, Nacalai Tesque, Japan); poly(dA)50 and poly(dT)50 single strand DNA (FASMAC Co., LTD, Japan); 1 kbp dsDNA (IDT); Forward and reverse primers (FASMAC); KOD SYBR® qPCR Mix (TOYOBO CO., LTD.); NucleoSpin® Gel and PCR Clean-up (Takara Bio Inc.); single strand DNA with and without G4 structure (Eurofins Genomics, Japan); potassium chloride (KCl, Nacalai Tesque, Japan); lithium chloride (LiCl, Nacalai Tesque, Japan); potassium hydroxide (KOH, FUJIFILM Wako Pure Chemical Industries); Tris(hydroxymethyl)aminomethane (Tris, Nacalai Tesque, Japan); hydrochloric acid (HCl, FUJIFILM Wako Pure Chemical Industries, Ltd. (Wako), Japan); alpha-hemolysin (αHL, Sigma-Aldrich, USA); poly-L-lysine hydrobromide (Mw=50,000~70,000, L-PLL, Sigma-Aldrich, USA) and poly(L-lysine hydrobromide) (Mw=10,000, S-PLL, Alamanda Polymers, USA). SV28 and SVG28 were synthesized and purified by Fmoc synthesis. DOPC was diluted to 10 mg/mL in *n*-decane. Buffered electrolyte solutions were prepared from ultrapure water, which was obtained from a Milli-Q system (Millipore, Billerica, USA). SV28 was dissolved at a concentration of 67.1 μM in ultrapure water and stored at −30 °C. SVG28 was dissolved at a concentration of 100 μM in ultrapure water: DMSO = 1 : 1(V/V) and stored at -30 °C.

1.2 MD simulation

The MD simulations of β-barrel structures of SV28 consisting of five and eleven peptides were performed in a DOPC membrane using GROMACS-5.1.4 and -2021.1[1] and CHARMM36 force field[2]. The 3D structures of the SV28 and SVG28 peptides were modeled *via* a homology modeling technique, which allows prediction of the structure of proteins based on the sequence similarity.[3] HASR protein (PDB ID: 3CSL; chain A; sequence positions, 484-511)[4] was selected as a template structure for the peptides from the Protein Data Bank using BLAST search.[5] The sequence covering and charged amino acid positions were applied as the main criteria for the template selection (**Fig. S1**). After modeling the β-barrel 3D structures of 5-mer, 7-mer, 11-mer, and 16-mer of SV28 and 7-mer SVG28 (**Figs. S3** and **S21**), the simulation systems were prepared by CHARMM-GUI membrane builder.[6] Then, the standard minimization, equilibration, and production procedures of the builder[6] were applied at 300 K temperature. Initially, 5-mer and 11-mer SV28 systems were equilibrated for 100 ns (**Fig. S1**). Finally, 900 ns MD simulations of 5-mer and 11-mer SV28 in DOPC membrane were performed under NPT conditions. 5-mer and 11-mer SV28 system were simulated for a total of 1 μs. The final simulations of the systems were analyzed and discussed. Additionally, MD simulations of 7-mer and 16-mer SV28 and 7-mer SVG28 pores were performed for 200 ns. The structures were represented by VMD software.[2] Analyses were performed using GROMACS packages, excluding the pore diameter, which was analyzed using HOLE software.[3]

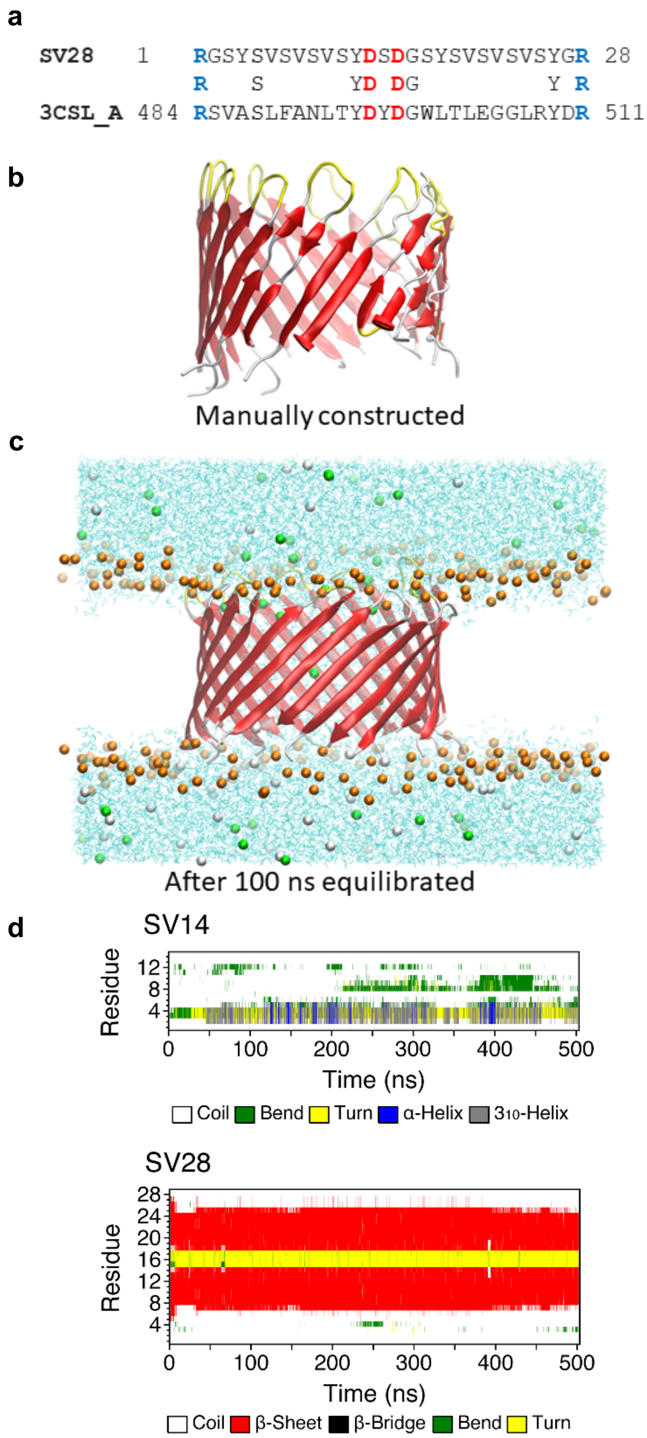

Fig. S1 (a) Sequence alignment of SV28 and HASR proteins (PDB ID: 3CSN, Chain A). Blue and red bold letters indicate the positively and negatively charged residues respectively. (b) A ribbon structure of the manually constructed initial model of 11-mer SV28. (c) An example of the 11-mer molecular system after a 100 ns long equilibration simulation. Red ribbons show the β-sheet regions of the structures with arrows pointing from the N-terminus to the C-terminus. (d) Secondary structure profiles of half- (upper) and full (lower) length of SV28 peptides during the MD simulations of the monomer.

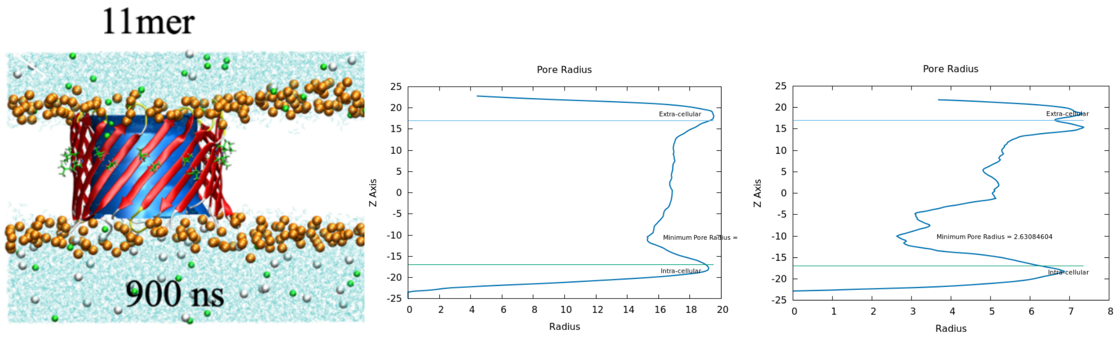

Fig. S2 Left figure shows the final snapshots of 11-mer SV28 in a DOPC membrane in the MD simulation. Ribbons show the peptide structures, with the secondary structure indicated by the color of the ribbon (red: β-sheet, cyan: turn, white: random coil structure). Ribbon arrows indicate the direction of the backbone from N-terminal to C-terminal. The pore radius of 5-mer and 11-mer were shown. The pore structures were analyzed by HOLE software and displayed as blue surfaces inside of the barrels. Val10 and Val22 amino acids showing central rim of the pores were displayed as the licorice models. Cyan lines indicate water molecules, and the lipid molecules were omitted for clarity (excluding phosphorus atoms as orange spheres). Green and white spheres indicate the potassium and chloride ions respectively. Structures were displayed by VMD software. The middle and right plots displayed the radius of the final snapshots of 11-mer and 5-mer pore, which were drown by CHARMM-GUI.

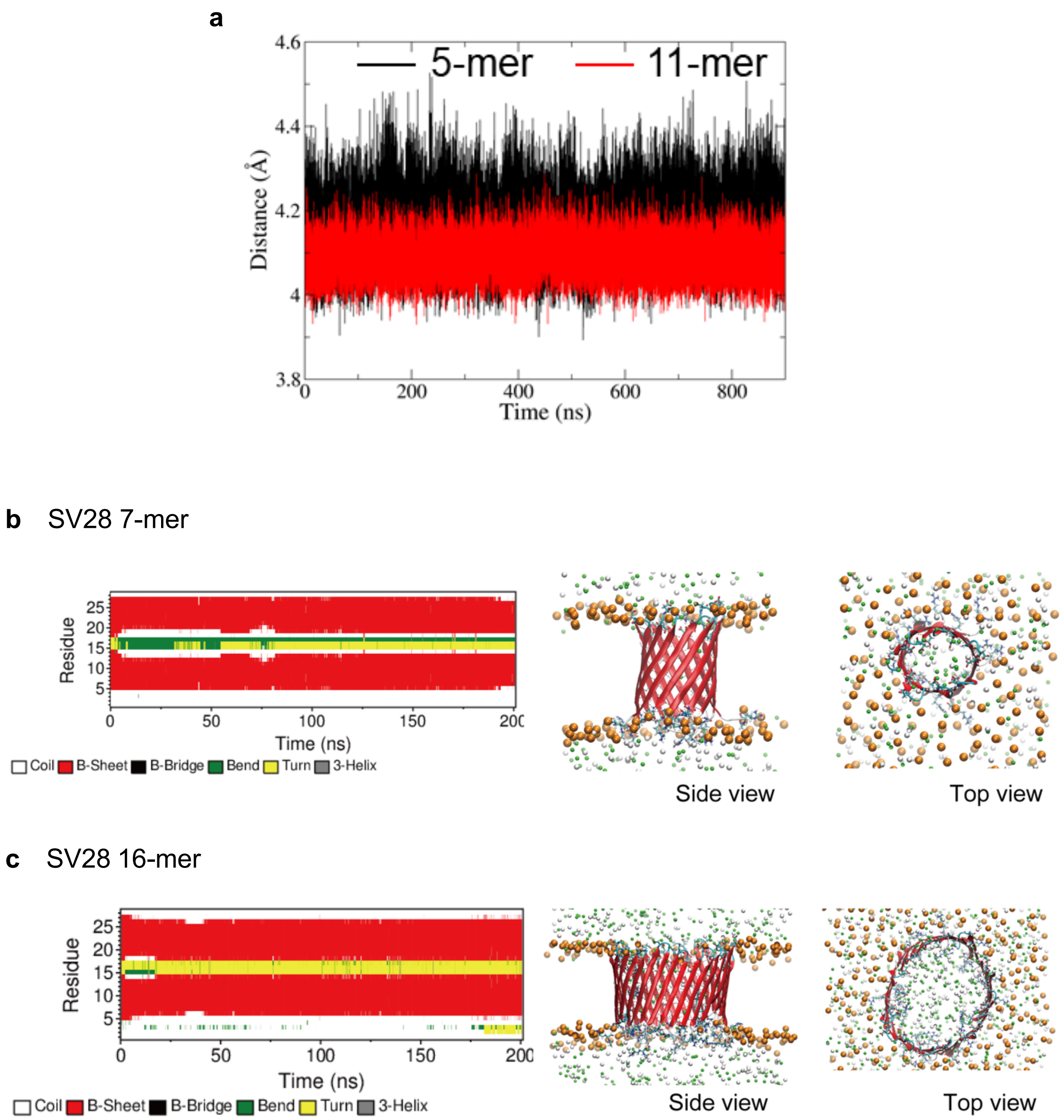

Fig. S3 (a) Intrapeptide distance between nitrogen of Val10 and carbon of Val22 during the simulations of 5-mer (black) and 11-mer (red). The distribution of the distance changing in the 5-mer was slightly larger than that in the 11-mer. 5-mer pore structures have shorter turns than 11-mer peptides, which can give structural distortion to the monomer structures. Because of this distortion, the distances in the 5-mer may become longer than that of 11-mer. (b) MD simulation of SV28 with 7-mer and 16-mer after 200 ns running.

1.4 Synthesis of Fmoc-1-^{13}C Tyr(tBu)-OH, Fmoc-2-^{13}C Gly-OH, and Fmoc-1-^{13}C Val-OH

Fmoc-1-^{13}C Tyrosine

1-^{13}C Labeled Tyrosine (1.15 g, 6.35 mmol) was dissolved in water (40 mL), followed by stepwise addition of Fmoc-OSu (3.71 g, 1.16 mmol) and $NaHCO_3$ (2.96 g, 3.45 mmol), with stirring for 1 day at room temperature. After the reaction mixture was neutralized with 5% hydrochloric acid, EtOAc was added, and the mixture was washed with saturated brine. The organic phase was dried over $MgSO_4$, and removed *in vacuo*. The residue was applied to a silica gel column and eluted with $CHCl_3$–MeOH (10:0.5) to give 2.35 g (75.7%) of the title compound as a white powder. ^{1}H NMR (500MHz, $CDCl_3$). δ 3.019 (2H, m), 4.1-4.5 (5H, m), 5.370 (1H, d, J = 8 Hz), 6.691 (2H, s), 6.919 (2H, s), 7.318 (2H, d, J = 7 Hz), 7.501 (2H, d, J = 7 Hz), 7.689 (2H, s), 8.320 (1H, br.s)

Fmoc-1-^{13}C Tyr-OCH_2CCl_3

To a solution of Fmoc-1-^{13}C Tyrosine (2.35 g, 5.81 mmol), 1-(3-Dimethylaminopropyl)-3-ethylcarbodiimide hydrochloride (EDC·HCl, 1.59 g, 6.97 mmol), and 2,2,2-trichloroethanol (2.77 ml, 29.1 mmol) in acetonitrile was added *N,N*-dimethyl-4-aminopyridine (DMAP, 83.6 mg, 0.58 mmol), and the mixture stirred for 4 hours at room temperature. After the reaction mixture was neutralized with 5% hydrochloric acid, the mixture was concentrated *in vacuo*. EtOAc was added, and the mixture was washed with saturated brine. The organic phase was dried over $MgSO_4$ and removed *in vacuo*. The residue was applied to a silica gel column and eluted with $CHCl_3$ to yield a quantitative amount of the title compound as an oil. ^{1}H NMR (500MHz, $CDCl_3$). δ 2.852 (3H, t), 3.05-3.205 (2H, m), 4.196 (1H, m), 4.298 (2H, m), 4.779 (2H, m), 6.754 (2H, d, J = 8 Hz), 7.014 (2H, d, J = 8 Hz), 7.302 (2H, dd, J = 7.5 and 8 Hz), 7.398 (2H, dd, J = 8 and 8 Hz), 7.543 (2H, dd, J = 7.5 and 8 Hz), 7.760 (2H, d, 7.5 Hz).

Fmoc-1-^{13}C Tyr(tBu)-OCH_2CCl_3

To a solution of Fmoc-1-^{13}C Tyr-OCH_2CCl_3 (3.99 g, 7.44 mmol) in 19 mL of super dehydrated tetrahydrofuran (Wako) was added boron trifluoride diethyl etherate (150 μL, 1.19 mmol), and a solution of tert-butyl 2,2,2-trichloroacetimidate (3.35 g, 14.9 mmol) in cyclohexane (19 mL) stepwise and the mixture stirred at room temperature. After the reaction mixture was neutralized with sodium carbonate powder, the filtered solution was concentrated *in vacuo*. The residue was applied to a silica gel column and eluted with $CHCl_3$ to give 1.58 g (35.8%) of the title compound as a white powder. ^{1}H NMR (500 MHz, $CDCl_3$). δ 1.20-1.40 (9H, m), 2.779 (1H, m), 3.10-3.20 (2H, m), 4.133 (2H, m) 4.388 (2H, m), 5.171(1H, br.s), 6.912 (2H, d, J = 8 Hz), 7.052 (2H, br.d, J = 8 Hz), 7.300 (2H, dd, J = 7.5 and 8 Hz), 7.392 (2H, dd, J = 8 and 8 Hz), 7.555 (2H, dd, J = 7.5 and 8 Hz), 7.755 (2H, d, 7.5 Hz).

Fmoc-1-^{13}C Tyr(tBu)-OH

Fmoc-1-^{13}C-Tyr(tBu)-OCH_2CCl_3 (1.05 g, 1.78 mmol) was dissolved in tetrahydrofuran (24 mL), and subsequently 50% aqueous acetic acid and zinc powder (5.14 g, 74.7 mmol) were added and stirred for 1 hour at room temperature. After filtration, ethyl acetate was added and the mixture washed sequentially with saturated aqueous sodium chloride and water. The organic phase was dried over $MgSO_4$ and removed *in vacuo*. The residue was applied to a silica gel column and eluted with a $CHCl_3$-MeOH (10:1) to give 306 mg (8.6%) of the title compound as a freeze-dried white powder. ^{1}H NMR (500 MHz, $CDCl_3$). δ 1319 (9H, s), 3.05-3.22 (2H, m), 4.203 (1H, br.t), 4.30-4,48 (2H, m), 4.665 (1H, br.s), 5.250 (1H, br.s), 6.913 (d, J = 8 Hz), 7.026 (2H, br.d, J = 8 Hz), 7.306 (2H, dd, J = 7.5 and 8 Hz), 7.399 (2H, dd, J = 8 and 8 Hz), 7.559 (2H, m), 7.763 (2H, d, 7.5 Hz).

Fmoc-2-^{13}C Gly-OH and Fmoc-1-^{13}C Val-OH

Fmoc-2-^{13}C Gly-OH and Fmoc-1-^{13}C Val-OH (99% isotopically enriched) were synthesized by the reaction of 9-fluorenylmethyl *N*-succinimidyl carbonate (Fmoc-OSu) with the ^{13}C labeled amino acids (Cambridge Isotope Laboratory CIL) based on the following reaction described by A. Paquet:[4] Fmoc-OSu (1 mmol) was reacted with one equivalent of the isotopically labeled amino acid in a water-acetone mixture in the presence of sodium bicarbonate (1 mmol). After stirring overnight at 30 °C, the mixture was acidified to pH 2 with concentrated HCl, and acetone was removed *in vacuo*. The product was dissolved in chloroform and washed with 0.1 N HCl and water. The organic phases were dried, and the residue was recrystallized by hexane-CH_2Cl_2. The melting points (m. p.) of Fmoc-2-^{13}C Gly-OH (174 °C) and Fmoc-1-^{13}C Val-OH (142 °C) were checked by a capillary method. The m. p. well agreed with the data[5].

1.5 Solid-phase synthesis of SV28, isotopic labeled SV28 and SVG28 precursors

Precursors of SV28, isotopic labeled SV28 A, B peptides (SV28-A, B) and SVG28 peptides (peptides before acyl migration, **Fig. S4**) were synthesized manually by traditional 9-fluorenylmethoxycarbonyl (Fmoc)-based solid-phase peptide method using Fmoc-NH-SAL-PEG resin (0.23 mmol/g, Watanabe Chemical Industries LTD. (Watanabe), Hiroshima, Japan). Peptide bonds were formed using 2-(1H-benzotriazole-1-yl)-1,1,3,3-tetramethyluronium hexafluorophosphate (HBTU, 10 eq., Watanabe) and 1-hydroxybenzotriazole monohydrate (HOBt, 10 eq., Watanabe) as a coupling reagent in the presence of Hünig's Base (*N*,*N*-diisopropylethylamine, 15 eq., Watanabe) for 30 min at 37°C. In the case of isotopic labeled Fmoc-amino acids (Fmoc-^{15}N Val-OH (Cambridge Isotope Laboratories, Inc., Tewksbury, MA, USA), Fmoc-2-^{13}C Gly-OH, Fmoc-1-^{13}C Val-OH, Fmoc-1-^{13}C Tyr(tBu)-OH), synthesis was performed using 1-

[bis(dimethylamino)methylene]-1*H*-1,2,3-triazolo[4,5-b]pyridinium 3-oxide hexafluoro-phosphate (HATU, 4 eq., Watanabe) in the presence of Hünig's Base (8 eq.) for 30 min at 37°C, and in the case of *O*-acyl isodipeptide [Boc-Ser(Fmoc-Val)-OH] (AAPPTec LLC, Louisville, KY, USA), synthesis was performed using N,N'-diisopropylcarbodiimide (DIPCDI, 4 eq., Watanabe), and HOBt (4.4 eq.) respectively[6,7] Each reaction was checked by Kaiser's test. Although Fmoc groups were removed using 25% piperidine in *N*-methylpyrrolidone (NMP, Wako), it is to be noted that Fmoc from the residue following the *O*-acyl isodipeptide units should be removed using Aimoto reagent[8], that is 25% 1-methylpyrrolidine (Tokyo Chemical Industry Co., Ltd. (TCI), Tokyo, Japan), 3% HOBt, and 2% hexamethyleneimine (TCI) in NMP-DMSO (Watanabe) (1:1), for 5min (5 times) at room temperature, because of the diketopiperazine formation. The final acetylation was performed using acetic anhydride(Watanabe) (20 eq.) in NMP in the presence of Hünig's Base (10 eq.). After the acetylation, peptidyl resins were washed with chloroform 5 times and were dried *in vacuo*. Cleavage from the resin and deprotection were performed by stirring with trifluoroacetic acid (TFA, Watanabe)/MilliQ/*m*-cresol (Wako)/thioanisole (Wako) (40/1/1/1, v/v/v/v, 5 mL) for 90 min[6,7] All the peptides were precipitated by the addition of cold diethyl ether, collected in 15 mL centrifuge tube by centrifugation (3000 rpm, 5 min, 4°C, CF15RXII (Koki Holdings Co.,Ltd.)) and dried *in vacuo*. Crude precursor peptides of SV28, SV28-A, SV28-B, and SVG28 were dissolved in 8 M guanidine hydrochloride (GuHCl) solution. The solutions were purified by preparative reverse phase HPLC (GL7410 pump and GL7450 detector system (GL Sciences Inc., Tokyo, Japan), column: Inertsil ODS-3, detection: UV 220 nm, elution: 0.1% TFA in water (A solution) and 0.08% TFA in an acetonitrile (B solution) gradient system; in the case of precursors of SV28, SV28-A and SVG28, isocratic condition with 100% of A

solution over 5 min, and then changing a gradient from 0% to 20% of B solution at next 5min, and then a linear gradient from 20% to 50% of B solution over 30 min at a flow rate of 3.0 mL/min (SV28, SV28-A, and SVG28 were eluted at 19.6 min, 19.9 min, and 20.1 min, respectively): in the case of the precursor of SV28-B and, isocratic condition with 100% of A solution over 5 min, and then changing a gradient from 0% to 15% of B solution at next 5 min, and then a linear gradient from 15% to 45% of B solution over 30 min at a flow rate of 3.0 mL/min (SV28-B was eluted at 26.2 min). Then the products were freeze-dried to give a white powder. MALDI-TOF MS: m/z: 2978.6 (calc. 2978.2) for SV28 precursor (total yield: 8.9 %, 50 μmol scale); m/z: 2982.1 (calc. 2981.2) for SV28-A precursor with (1-^{13}C) labeled ^{12}Y, (2-^{13}C) labeled ^{16}G and ^{15}N labeled ^{20}V (total yield: 7.4 %, 25 μmol scale) and 2982.3 (calc. 2981.2) for SV28-B precursor with (1-^{13}C) labeled ^{10}V, (2-^{13}C) labeled ^{16}G, N^{15} labeled ^{22}V (total yield: 8.9 %, 10 μmol scale); m/z: 2919.8 (calc. 2919.6) for SVG28 precursor (total yield: 14.7 %, 25 μmol) (**Fig. S5**). The peptides were stored at 4°C. Before the following measurements, purified peptides except for SVG28 were dissolved in MilliQ water to approximately 1 mM and SVG28 was dissolved in methanol (MeOH) to approximately 1 mM (due to easy aggregation and very poor solubility in MilliQ water). Then their concentrations were determined by amino acid analysis.

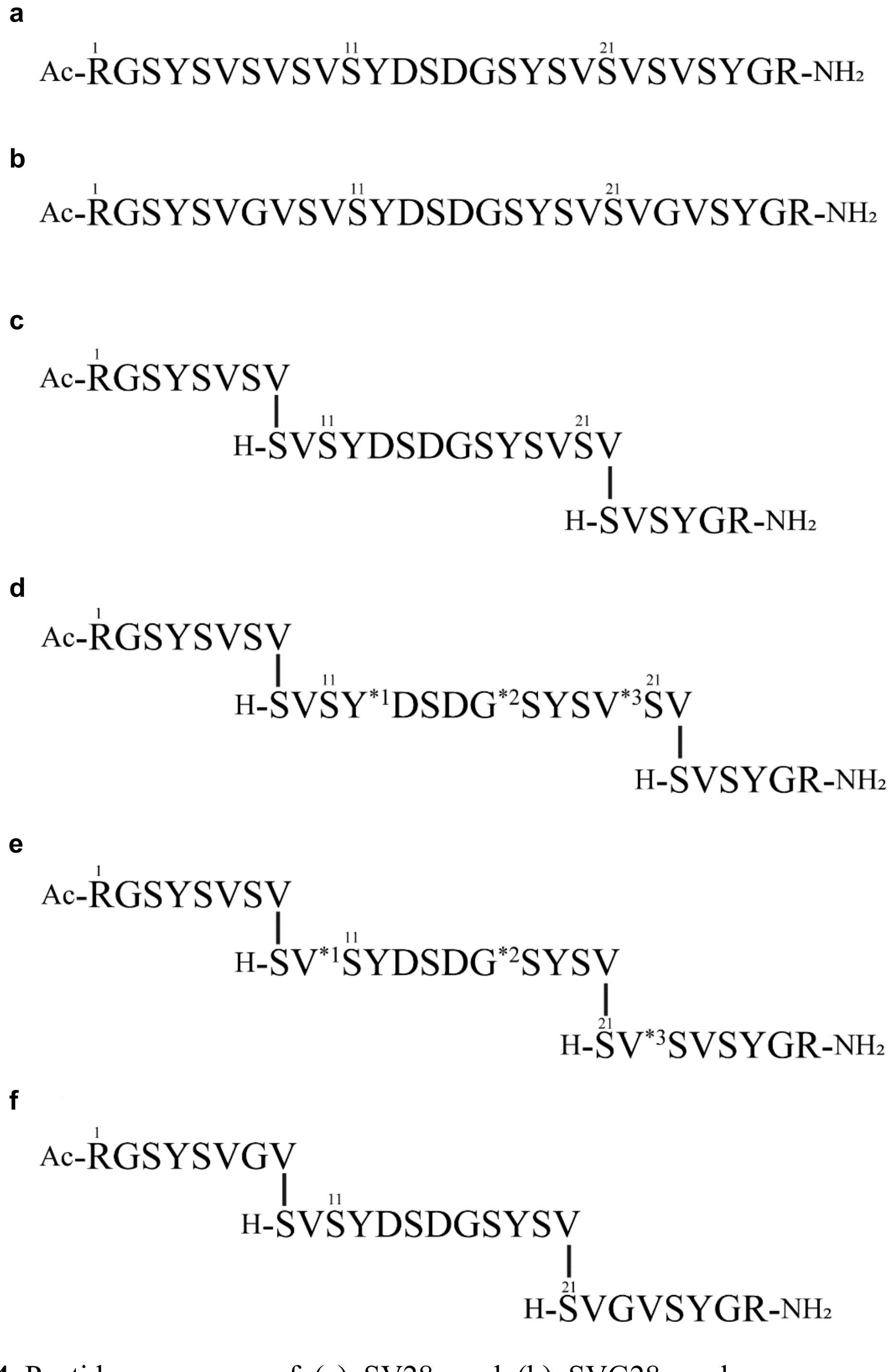

Fig. S4 Peptide sequences of (a) SV28, and (b) SVG28, and precursor sequences (peptides before acyl migration) of (c) SV28, (d) isotopic labeled SV28-A with (1-^{13}C) labeled ^{12}Y, (2-^{13}C) labeled ^{16}G and ^{15}N labeled ^{20}V (*^{1} (1-^{13}C) labeled Y, *^{2} (2-^{13}C) labeled G, *^{3} ^{15}N labeled V), (e) isotopic labeled SV28-B with (1-^{13}C) labeled ^{10}V, (2-^{13}C) labeled ^{16}G, ^{15}N labeled ^{22}V (*^{1} (1-^{13}C) labeled V, *^{2} (2-^{13}C) labeled G, *^{3} ^{15}N labeled V) and (f) SVG28.

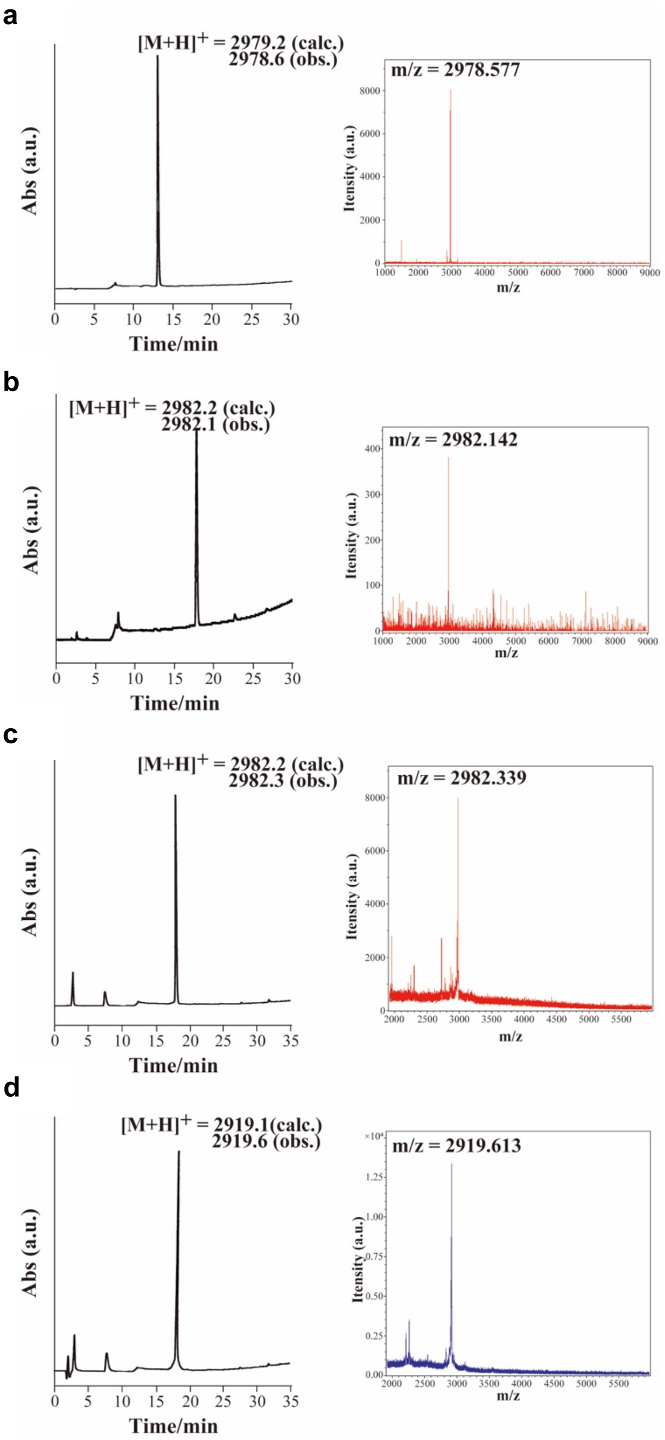

Fig. S5 HPLC and MS spectra for purified peptides precursor of (a) SV28, (b) SV28-A, (c) SV28-B separated and (d) SVG28 on an ODS column (150 x 4.6 mm, 5 µm) with (a), (b) A solution using a gradient from 0% to 100% B solution over 30 min [(c), (d) gradient started after 5 minutes], 1.0 mL/min, detection at 220 nm.

1.6 Confirmation of acyl migration by HPLC

The acyl migration of the peptides was confirmed by checking the decrease in the peak area of precursor peptides in HPLC (**Fig. S6**) because the final products after acyl migration were easily aggregated and were hardly eluted in standard HPLC condition. The precursors of SV28, SV28-B, and SVG28 were added to 100 mM KOH and incubated for 5 minutes to allow acyl migration of isoacyl dipeptide moiety to the native Val-Ser dipeptide moiety. Then, in the case of SV28 and SV28-B, 1 M HCl was used for neutralization, and 30 mM MOPS buffer and MilliQ were added to the sample, and in the case of SVG28, 1M HCl was used for neutralization, and 30 mM MOPS buffer and MeOH were added to the sample (50% MeOH). The samples were incubated for 0 h or 24 h at 37°C and then analyzed by HPLC.

The peptides were analyzed by absorbance at 220 nm on an Inertsil ODS-3 column (150 x 4.6 mm, 5 μm, GL Sciences Inc.) using isocratic condition with 100% of MilliQ (containing 0.1% TFA) over 5 minutes, and then a linear gradient from 0% to 100% acetonitrile (containing 0.08% TFA) over 30 min at a flow rate of 1.0 mL/min (**Fig. S6**).

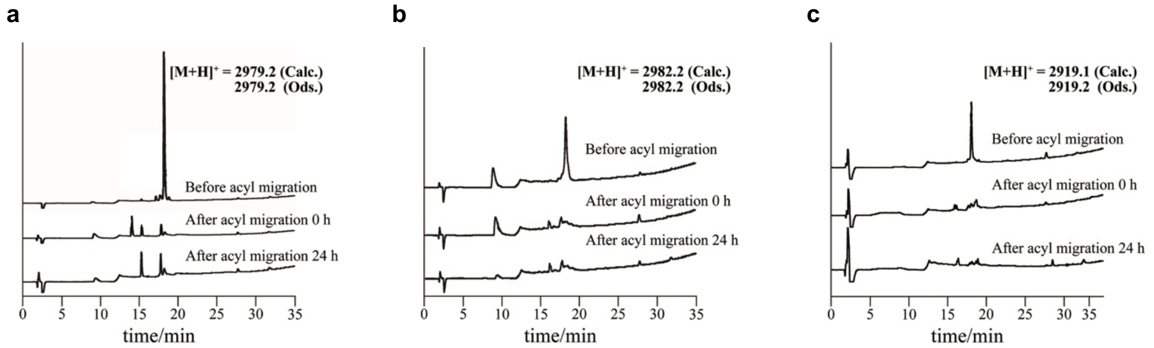

Fig. S6 HPLC chromatogram and MALDI-TOF MS of the sample before and after acyl migration by using (a) SV28, (b) SV28-B, and (c) SVG28. MALDI-TOF MS results of the samples before acyl migration could be determined and were shown in the figure. In contrast, the results of the samples after acyl migration could not be determined because the precursors' peaks did not emerge in HPLC (the precursors were completely acyl migrated).

1.7 Liposome preparation for CD measurements

A 20× liposome stock solution (10 mM DOPC in 10 mM MOPS, pH 7.0) was made by the gentle hydration method. DOPC 31.4 µL (50 mg/mL in $CHCl_3$) was added to the vial bottle. $CHCl_3$ was vaporized under N_2 gas flow and a vacuum desiccator for 3 hours to form a lipid film at the bottom of the bottle. In the case of SV28 and SV28-B, 10 mM MOPS buffer was added to the vial bottle and the solution was sonicated at 50 °C for 30 s. In the case of SVG28, 10 mM MOPS buffer and MeOH were added to the vial bottle (50% MeOH) and the solution was sonicated at 50 °C for 30 s.

1.8 Measurements of CD spectroscopy

CD spectroscopy was performed at room temperature using the synthesized peptides. In

the case of the migrated SV28 and SV28-B, 50 μM peptides were incubated for 0 hours, or 24 hours at 37°C in MilliQ water, 1 M KCl, and 10 mM MOPS with 50 μM DOPC liposome. In the case of migrated SVG28, 50 μM peptides were incubated for 0 hours, or 24 hours at 37°C in MeOH, 1 M KCl, and 10 mM MOPS (pH 7.0, 50 % MeOH solution) with 50 μM DOPC liposome. CD spectroscopy was conducted on the 20-fold diluted solutions of SV28 and SV28-B by MilliQ or the 2-fold diluted solution of SVG28 by MilliQ and MeOH (1/1, v/v) for 5 min (molar ratio; 1:20 (peptide : lipids)). A J-820 spectropolarimeter (JASCO) with a thermoregulator at 25 °C and a quartz cell with a 1 cm path length was used. Each sample was scanned four times with the average of spectra reported. (**Fig. S7**)

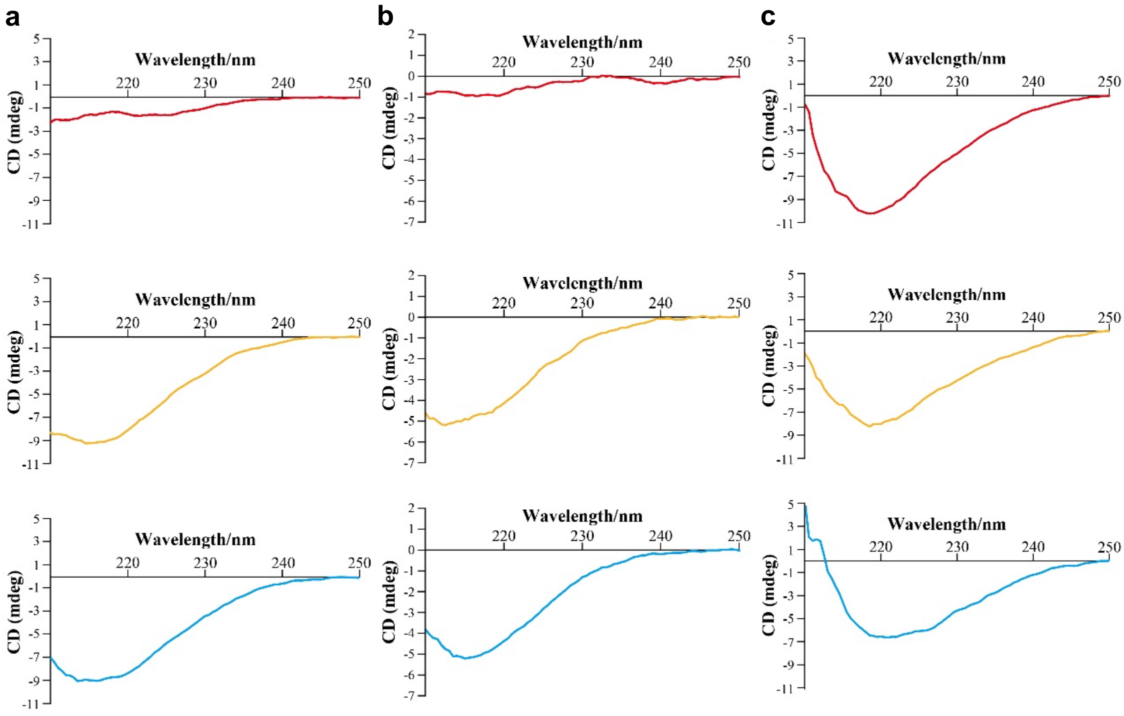

Fig. S7 CD spectra with DOPC liposome before and after acyl migration using precursors of (a) SV28, (b) SV28-B, and (c) SVG28. (red line: before migration, yellow line: after migration 0 h, blue line: after migration 24 h).

1.9 Solid-state NMR measurements

The synthesized SV28-B ([1-^{13}C]Val10, [2-^{13}C]Gly16, [^{15}N]Val22-labeled SV28) was reconstituted into DOPC liposome in a buffer (10 mM MOPS, mM KCl, pH 7.0) as a molar ratio of 1:25 (peptide : lipids). The hydrated liposome samples were directly packed into a 4.0 mm outer diameter zirconia rotor. ^{13}C and ^{15}N cross-polarization magic angle spinning (CP-MAS) NMR experiments were performed on a 600 MHz solid-state NMR spectrometer (Bruker Avance III) equipped with a ^{1}H-^{13}C-^{15}N triple resonance E-free MAS probe at the temperature 277 K with a contact time of 1.0 and 1.5 ms, and recycle time of 3.0 s. MAS frequency was set at 10.0 kHz during the measurements. REDOR and full echo experiments were performed to determine the ^{15}N-^{13}C internuclear dipolar interaction.[9] Here, ^{15}N echo signals were detected because of smaller contributions from natural abundant ^{15}N nuclei (0.37%). The error of the flip angle was compensated using the REDOR sequence, the *xy* eight-pulse program for irradiation of ^{13}C nuclei to recouple the ^{15}N-^{13}C dipolar interaction. The temperature and MAS frequency were set at 263 K and 4.0 kHz. ^{15}N REDOR (S) and full echo ($S_{fullecho}$ =S_0) spectra were obtained at various dephasing times NcTr (Nc is the number of rotor cycles and Tr is the rotor cycle period). The normalized REDOR difference was obtained as $\Delta S/S_0$. Plots of $\Delta S/S_0$ against NcTr reflect the ^{15}N-^{13}C dipolar interaction and thus ^{15}N-^{13}C internuclear distance information as well. ^{13}C chemical shifts were externally referenced to adamantane at 40.48 ppm (DSS: 0.0 ppm). ^{15}N chemical shifts were externally referenced to $^{15}NH_4Cl$ at 38.44 ppm.

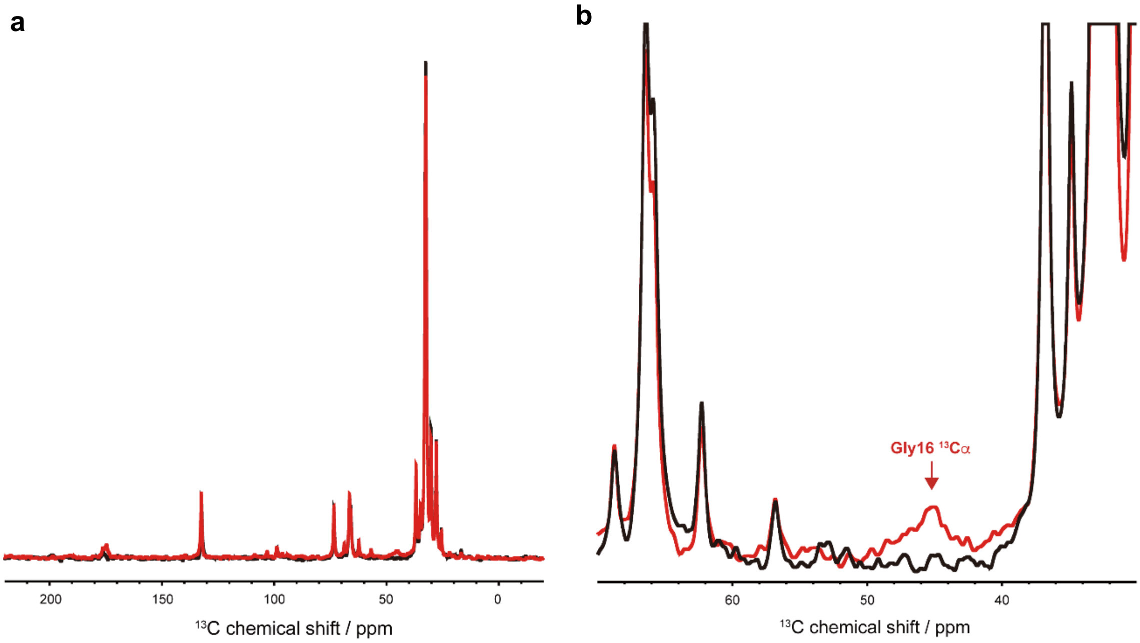

Fig. S8 Solid-state NMR spectra. (a, b) ^{13}C CP-MAS NMR spectra of the triply isotope-labeled SV28 ([1-^{13}C]Val10, [2-^{13}C]Gly16, [^{15}N]Val22-labeled SV28) in DOPC liposomes. The black lines indicate the spectra from DOPC liposomes, and the red lines indicate the spectra of isotope-labeled SV28 with DOPC liposomes.

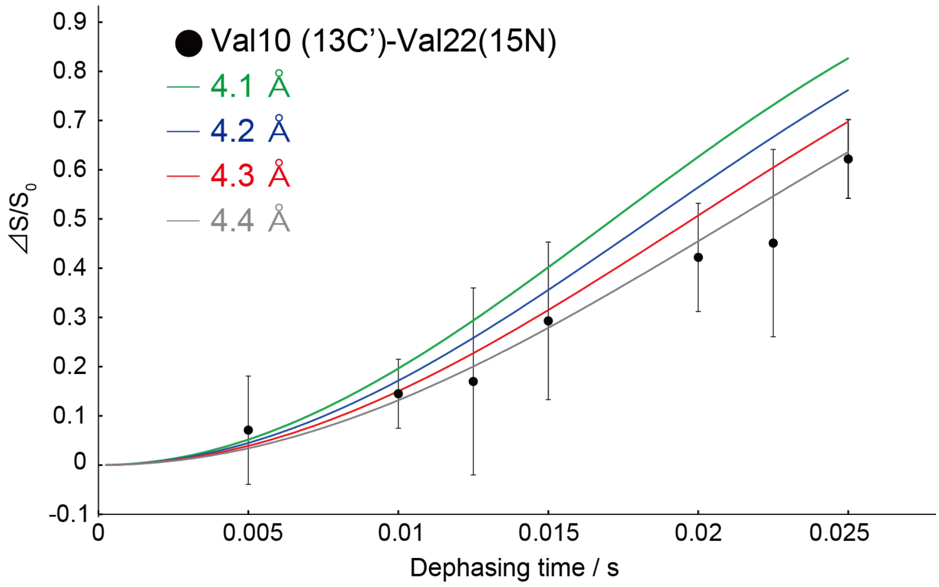

Fig. S9 REDOR difference plot of SV28 Val22(^{15}N)-Val10(^{13}C') by observation of the ^{15}N peak intensity. (S) and (S_0) are recorded under REDOR and full echo conditions. REDOR curves correspond to an isolated ^{15}N-^{13}C pair with varying distances (green, blue, red, and gray curves correspond to 4.1, 4.2, 4.3 and 4.4 angstroms). The distance between the backbone amide of Val22 and the carbonyl carbon of Val10 is estimated to be around 4.4 Å.

1.10 *Fabrications of the microdevices of lipid bilayer system*[10]

Microdevices were fabricated by machining a 6.0 mm thick, 10 × 10 mm polymethyl methacrylate (PMMA) plate (Mitsubishi Rayon, Tokyo, Japan) using computer-aided design and manufacturing by a three-dimensional modeling machine (MM-100, Modia Systems, Japan) as shown in **Fig. S7b**. Two wells (2.0 mm diameter and 4.5 mm depth) and a chase between the wells were manufactured on the PMMA plate. Each well had a through-hole in the bottom and Ag/AgCl electrodes set into these holes (**Fig. S7c**). A polymeric film made of parylene C (polychloro-*p*-xylylene) with a thickness of 5 μm was patterned with single pores (100 μm diameter.) using conventional photolithography methods,[11] and then fixed between PMMA films (0.2 mm thick) using an adhesive bond (Super X, Cemedine Co., Ltd, Tokyo, Japan). The films, including the parylene film, were inserted into the chase to separate the wells. High throughput measurement (**Fig. S7d**) was conducted using a JET patch-clamp amplifier (Tecella, Foothill Ranch, CA, USA).[12]

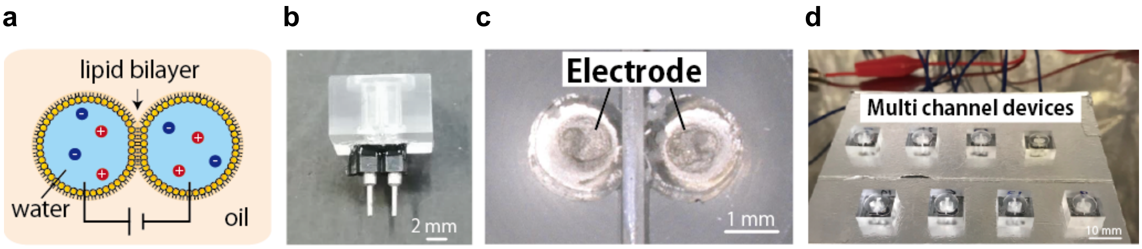

Fig. S10 Fabricated microarray device for a lipid bilayer system. (a) Schematic illustration of the droplet contact method. (b) A photograph of a microdevice for the droplet contact method and (c) its enlarged picture. (d) A photograph of a multiarray device that can form eight individual lipid bilayers simultaneously.

1.11 Preparation of bilayer lipid membrane and alpha-hemolysin pretreatments

BLMs were prepared in the same method as above using DPhPC (lipids/*n*-decane, 10 mg/mL) solution. The buffer solution (4.7 μL) with αHL (final concentration 50 nM) was poured into the recording chamber. The buffer solution (4.7 μL) was poured into the ground chamber. In this experiment, a buffer solution (1 M KCl, 10 mM MOPS, pH 7.0) was used.

2. Channel current measurements and data analysis

Channel current was monitored using a JET patch-clamp and PICO2 amplifier (Tecella, USA) connected to the chambers. Ag/AgCl electrodes were already present in droplets when the solution was added to the chambers. A constant voltage ranging from 40 to 200 mV was applied to the recording chamber, and the other chamber was grounded. Pore formation in BLMs allowed ions to pass through the nanopore under the voltage gradient, giving the channel current signals. The signals were detected using a 4 kHz low-pass filter at a sampling frequency of 20 kHz. Analysis of channel current signals and duration time was performed using pCLAMP ver. 11.0.3 (Molecular Devices, USA), Excel (Microsoft, USA) software, and in-house programs coded by a python. Channel current measurements were conducted at 22 ± 2 °C. We assessed the relationship between the current conductance and the pore diameter of β-barrel proteins, measurements that were taken by electrophysiology, and by crystallography using microscopes. OmpA[13], OmpF[14], OmpG[15], VDAC[16], and FhuA[17] were used in this estimation as the β-barrel transmembrane proteins. The calibration curves (**Fig. 3g** and **3h**) were prepared for representing the relationships between the channel conductance and the number of β-strands (**Fig. 3g)** and between the pore diameter and the number of β-strands (**Fig. 3h**).

Using these calibration curves, we estimated the pore diameter and the number of β-strands of the SV28 and SVG28. We set a threshold conductance form the open level to determine the single-molecule detection in the channel current recoding: SV28-dsDNA 1nS (**Fig. 4a**); SV28-G4 0.5 nS (**Fig. 4i** and **4j**); SVG28-PLL 0.4 nS (**Fig. 5f**); αHL-PLL 0.2 nS (**Fig. 5l** and 5m). Peaks of conductance histograms (**Fig. 3f** and **Fig. 5c**) were founded by 2nd derivative method (threshold: 15% of the counts) and fitted by nonlinear and Gaussian curve fitting method (see the detail on the website of OriginLab: https://www.originlab.com/index.aspx?go=products/origin/dataanalysis/peakanalysis) using OriginPro8.5j (Light Stone, Japan).

We considered how SV28 forms a nanopore with a β-barreled structure. There have been many reports on the pore/channel formation of α-helical peptides. These peptides initially bind to the surface of the lipid membrane and form α-helical structures, which subsequently assemble from the monomers to construct the transmembrane nanopore structure. Several pore-forming models have already been proposed for these structures, such as barrel-stave or toroidal models.[18] We have also proposed the assignment of current signals to these models in planar lipid bilayer experiments.[19] Although there are few studies on the pore-formation of β-sheet peptides, it has been reported that the β-sheet peptides also construct barrel-stave and toroidal pores.[20] Our electrophysiological measurements herein also display step and multi-level signals, analogous to those previously assigned to the barrel-stave and the toroidal models.

Although we used experimentally estimation of the pore diameter, the pore diameter of the SV28 nanopore was also calculated using the conductance of the open channel state and the Hille equation, which is a theoretical model that uses the resistance of a cylindrical pore to ion flow.[21] The open channel conductance was determined as the initial step signal

from the baseline (≈0 A). The histogram of the pore conductance of SV28 is shown in **Fig. 3f.** Several peaks are observed in this histogram, with five identified peaks picked by the second deviation method. The peak conductance at 1, 3, 7, 11, and 14 nS are identified, and these give pore diameters of 0.8, 1.7, 2.3, 2.7, and 3.5 nm respectively using the Hille model. The numbers of monomers used in the nanopore assembly were mathematically calculated to be 4, 5, 7, 8, and 10 monomers using the diameters and the size of the β-hairpin molecule.

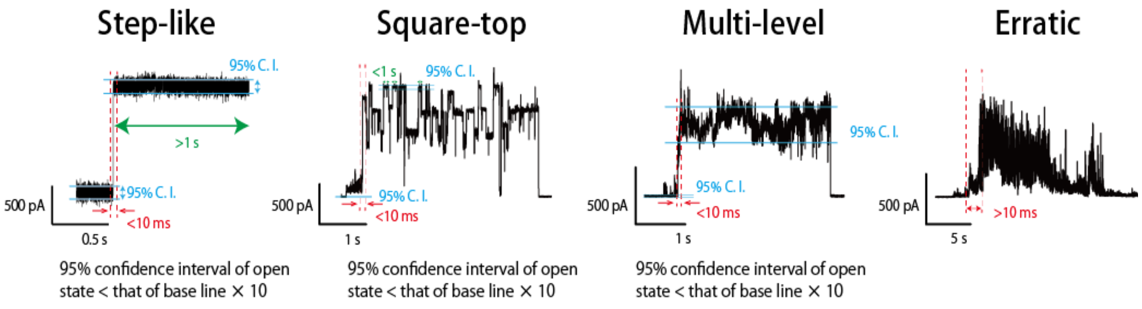

Fig. S11 The definition of current signal classification. Step-like signal: the current sharply increases (within 10 ms) and maintains a plateau state (longer than 1 s). Square-top signal: the current sharply increases (within 10 ms) and proceeds to transit plateau states (shorter than 1 s). Multi-level signal: the current sharply increases (shorter than 10 ms) and proceeds to fluctuate. Fluctuation defined as when the 95% confidence interval of the open level current is larger than that of the baseline. Erratic signal: the current randomly increases with fluctuation.

2.1 Preparation of dsDNA with 1 kbp and ssDNA with and without G4 DNA

Two kinds of double-stranded DNA were prepared: 50 bp and 1 kbp dsDNA were prepared by annealing dA50 and dT50. 1 kbp dsDNA, a part of lambda DNA (9346-10345: 1kbp), was purchased and amplified using polymerase chain reaction (PCR). PCR solution contained KOD SYBR® qPCR Mix (half of the total volume), template DNA (75 fM), and forward primer (1 μM), and reverse primer (1 μM). Then, the amplified dsDNA was purified by NucleoSpin® Gel and PCR Clean-up. The concentration of purified dsDNA was measured by the absorbance at 260 nm using NanoDrop 2000c. The annealed dsDNA was added to buffer solution in a grand chamber before adding the solution to the recording chamber. The threshold of DNA translocation was decided as blocking of more than 1 nS from the open-pore current level as in a previous study.[22] As for the G4 detection, the buffer solution (4.7 μL) with 24 hours incubated SV28 peptide (final concentration 1 μM) and single-strand DNA (sequence as shown in table, final concentration 2 μM) were poured into the ground chamber. In this study, a buffer solution (50 mM KCl, 950 mM LiCl, 25 mM Tris, pH 7.9) was used.

With G4 structure	A_{38}-(TTAGGG)$_{20}$-TT
Without G4 structure	A_{38}-(TTATCG)-(TTATCA)$_{18}$-(TTATCG)TT

2.2 Preparation of bilayer lipid membrane and SVG28 pretreatments

SVG28 was dissolved at a concentration of 100 μM in ultrapure water:DMSO = 1:1(V/V) and stored at -30 °C. αHL was dissolved at a concentration of 31 μM in ultrapure water and stored at -30°C. L-PLL and S-PLL were dissolved at a concentration of 1 mM and 5 mM respectively in ultrapure water.

Bilayer lipid membranes (BLMs) were prepared by the droplet contact method using a microdevice. First, the DOPC: cholesterol = 4 : 1 (w/w) (lipids/*n*-decane, 10 mg/mL) solution (0.6 μL) was poured into each chamber. Next, the buffer solution (4.7 μL) without any peptide was poured into the recording chamber. The buffer solution (4.7 μL) with peptide (final concentration 1 μM) was poured into the ground chamber. In this study, a buffer solution (1 M KCl, 10 mM MOPS, pH 7.0) was used. Before the measurement, the peptides were added to 100 mM KOH and incubated for 5 minutes in order to allow the transfer of isoacyl dipeptide to the native dipeptide of **Val** and **Ser**. Then, HCl was added to make the buffer pH 7. A few minutes after adding the buffer solution, the two lipid monolayers connected to form BLMs. When the BLMs ruptured, they were reconstituted as BLMs by tracing with a hydrophobic stick between two droplets. The solutions were prepared comprising of 2 μM transformed SVG28, 1 M KCl, 10 mM MOPS, and 10 mg/mL DOPC: cholesterol = 4 : 1 (w/w) in *n*-decane at pH 7. The solution was agitated in a vortex for 30 s and incubated for 24 h at 37°C. The lipid and buffer solutions were added to the ground chamber.

2.3 Detection of poly-L-lysine

In the detection of poly-L-lysine, we used long-poly-L-lysine (L-PLL) which has 132~300 amino acids, and short-poly-L-lysine (S-PLL) which has 50 amino acids. Both L-PLL and S-PLL were added to the buffer solution in a recording chamber. In detection using SVG28, the buffer solution with 24 hours incubated SVG28 was added in a ground chamber. In detection using αHL, the buffer solution with αHL (final concentration 50 nM) was added in a recording chamber with a DPhPC bilayer. The threshold of PLL translocation using SVG28 and αHL was decided as blocking of more than 0.4 and 0.2

nS respectively from the open-pore current level. In the exact bootstrap method, the verification of accuracy will be made possible when the sample number is over 200. In this study, our bootstrap procedure took 1000 or 300 samples randomly from the primary common translocation data with 65536 replacements and to calculate the means for these samples.

3. Single-molecule detection using SV28 and SVG28 nanopore (**Fig. S12 – S19**)

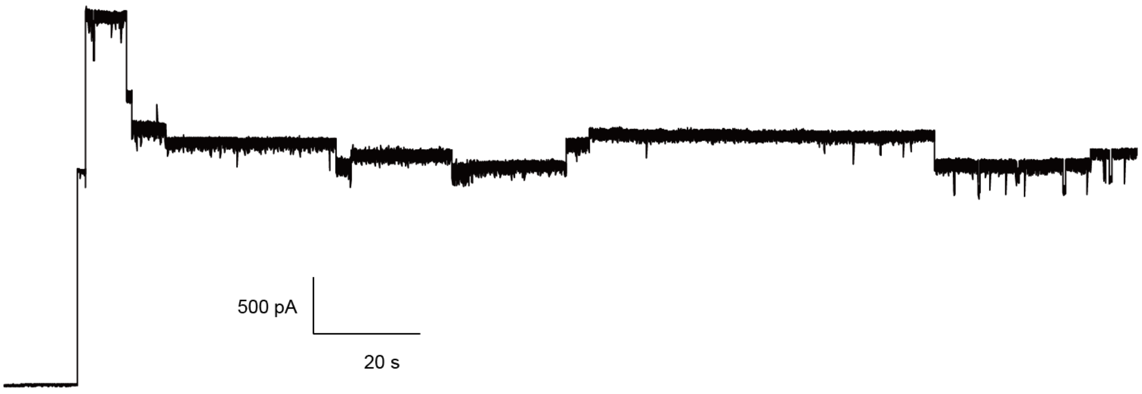

Fig. S12 The typical current and time trace of SV28. The signal was observed under optimized conditions: 1 μM SV28 (24 hours incubation at 37°C with DOPC and 20 % cholesterol), 1 M KCl, 10 mM MOPS, and an applied voltage of +200 mV.

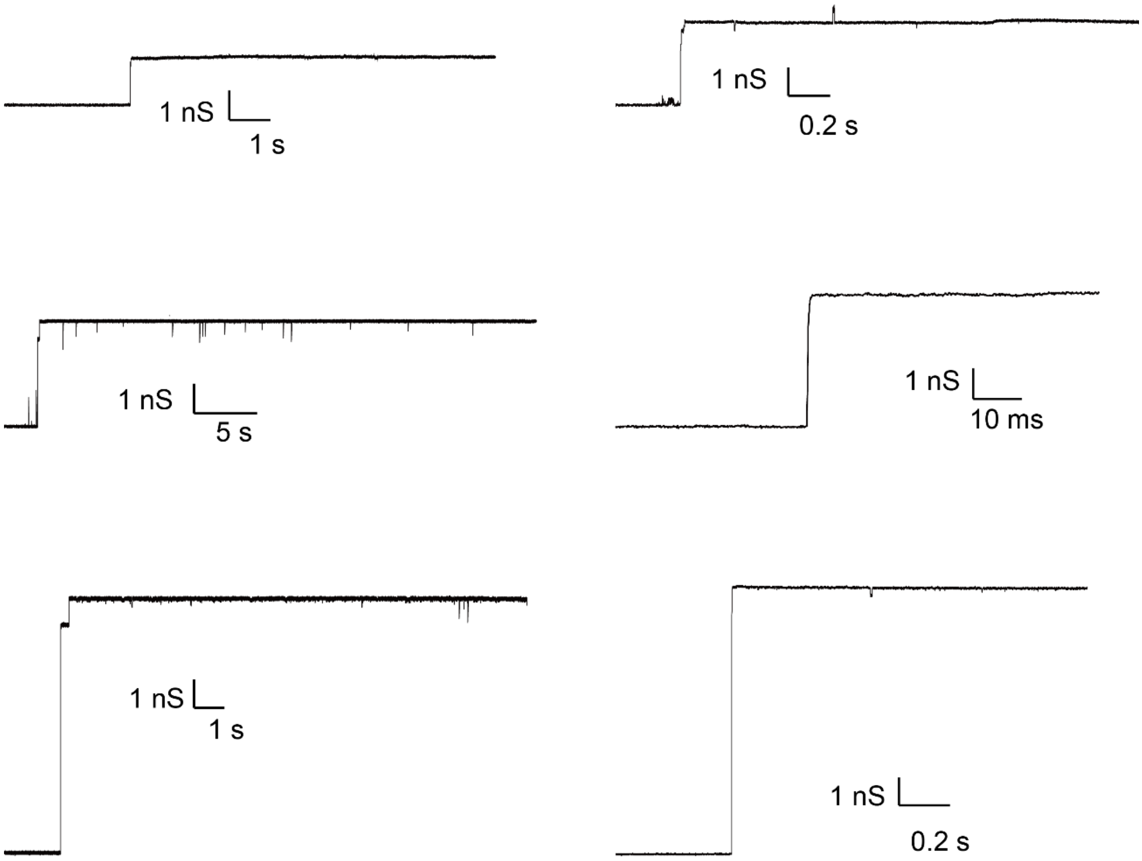

Fig. S13 Several conductance and time traces of the initial step signals of SV28. These step signals rose up from the baseline. These step signals were observed under the following conditions: 1 μM SV28 (24 hours incubation at 37°C with DOPC and 20 % cholesterol), 1 M KCl and 10 mM MOPS. The applied voltages were +120 mV or +200 mV.

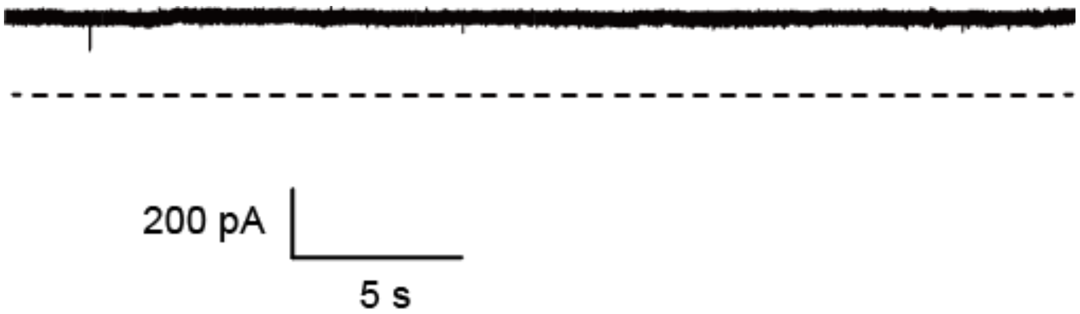

Fig. S14 The open level of the step signal of SV28 with 10 μM 50 bp dsDNA (short dsDNA). The dashed line indicates the baseline level. The current signal was measured under optimized conditions: 1 μM SV28 (24 hours incubation at 37ºC with DOPC and 20 % cholesterol), 1 M KCl, 10 mM MOPS, and an applied voltage of +200 mV.

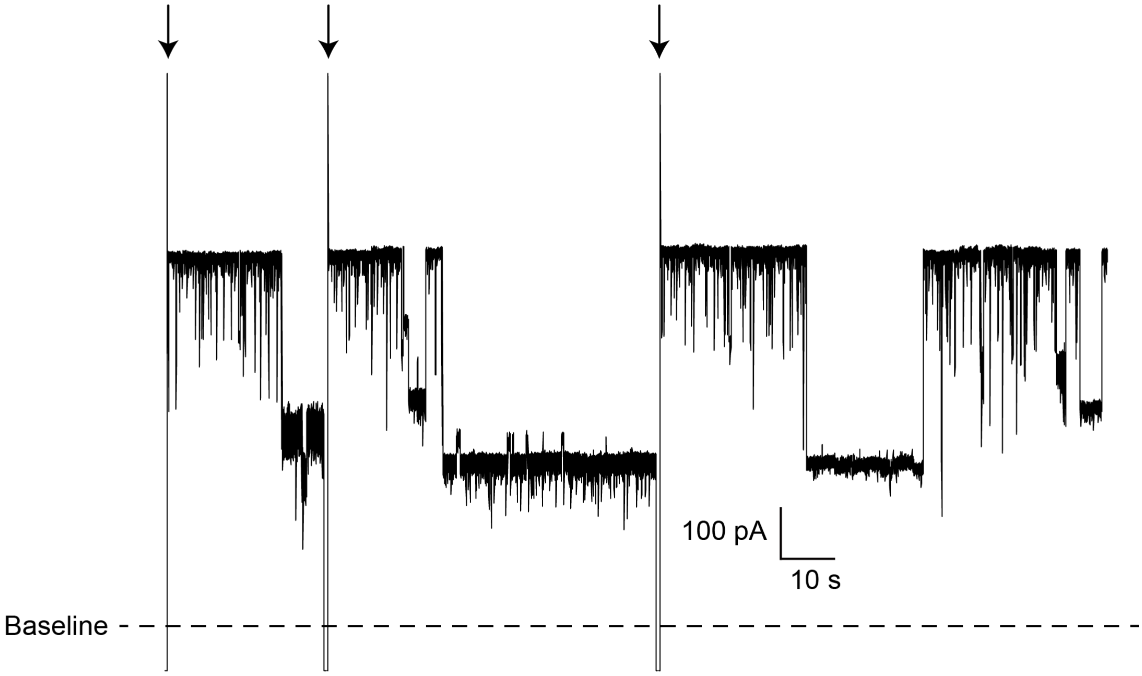

Fig. S15 Long time (3 minutes) traces of dsDNA translocation into SV28 nanopore. The long and deep blocking currents sometimes observed might be clocking of dsDNA in the nanopore, so the long and deep blocking were released by voltage transitions which were shown as the arrows. The trace was observed under below conditions: 1 μM SV28 (24 hours incubation at 37°C with DOPC and 20 % cholesterol), 1 M KCl, 10 mM MOPS, an applied voltage of +100 mV, and 100 nM dsDNA with 1 kbp.

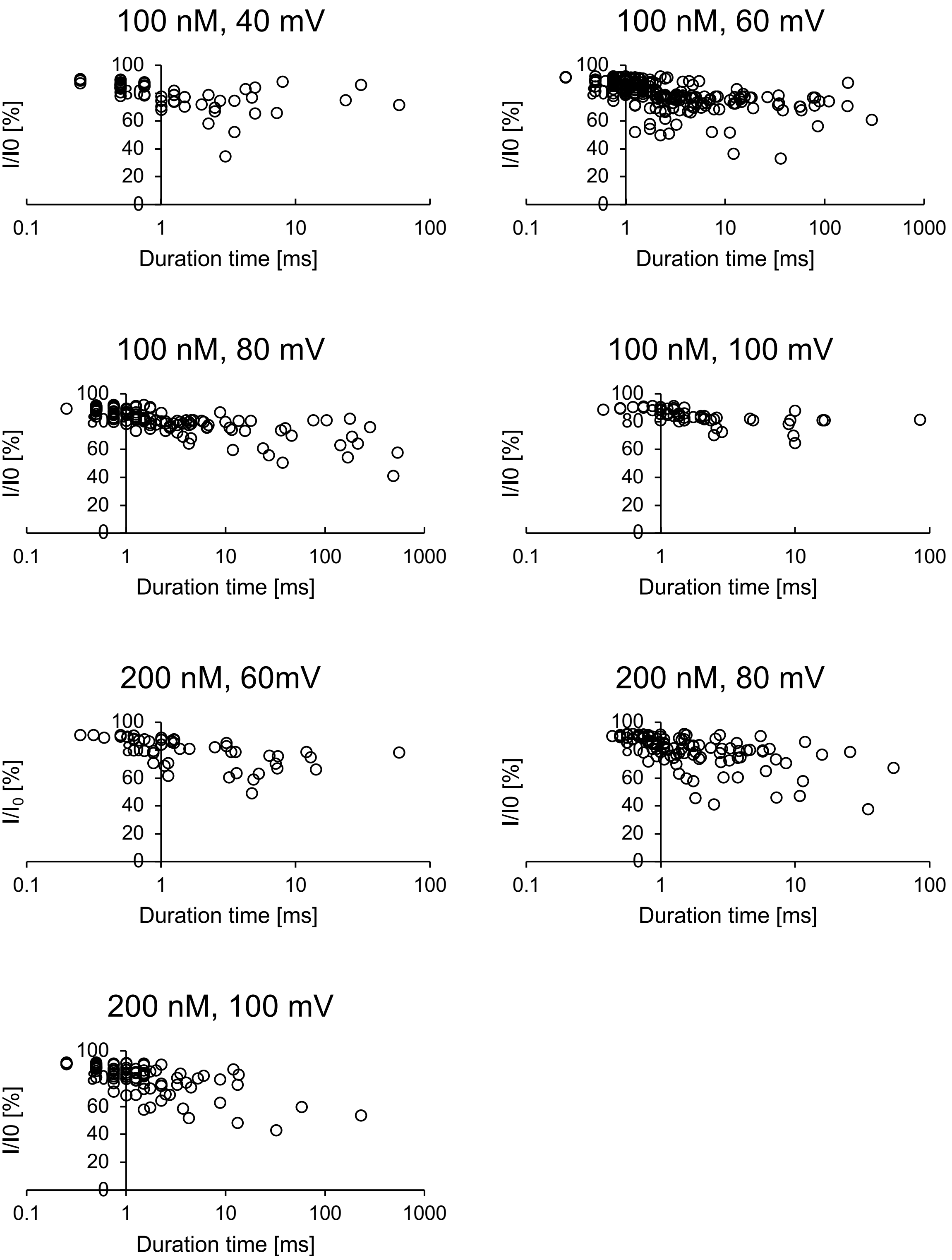

Fig. S16 Scatter plots of percent current blockage and duration for 1 kbp dsDNA translocation through an SV28 pore with diameter of around 5 nm. The typical current signal was observed under the following conditions: 1 µM SV28 (24 hours incubation at 37°C with DOPC and 20 % cholesterol), 1 M KCl and 10 mM MOPS.

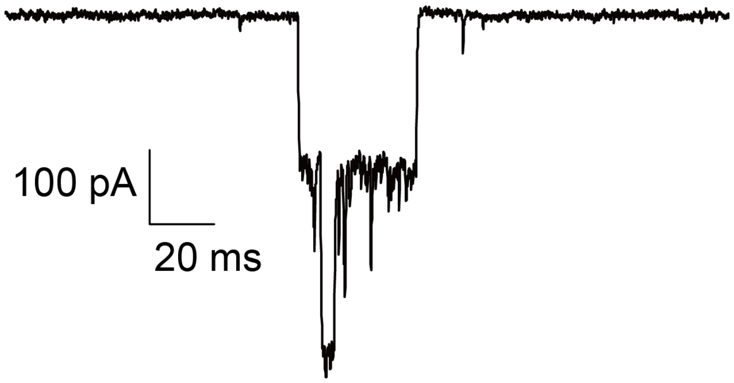

Fig. S17 Typical overlapping blocking currents. Two different current levels were occasionally observed, probably due to translocation of multiple dsDNA though the SV28 nanopores simultaneously. The current signal was observed under the following conditions: 1 μM SV28 (24 hours incubation at 37°C with DOPC and 20 % cholesterol), 100 nM dsDNA, 1 M KCl, 10 mM MOPS and an applied voltage of +60 mV.

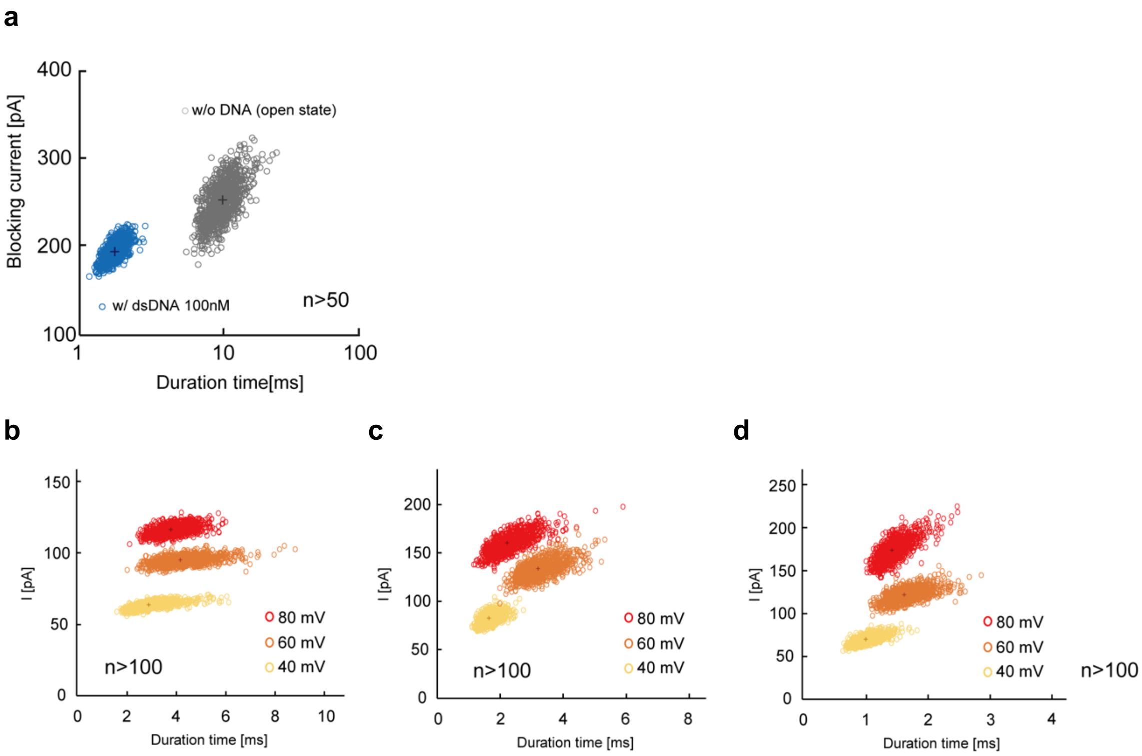

Fig. S18 Scatter plots of the duration and blocking current of dsDNA (1 kbp) through the SV28 nanopore (5 nm diameter) after bootstrapping. (a) The scatter plots comparison between presence and absence dsDNA at 100 mV. There are inherent current blockings in the pore open state in SV28 shown as gray plots. (b-d) Scatter plots of duration and blocking current of dsDNA (1 kbp) through SV28 nanopore (5 nm dia.) depended on the concentration of (b) 50 nM, (c) 100 nM, and (d) 200 nM under different voltage applications (40 mV to 80 mV).

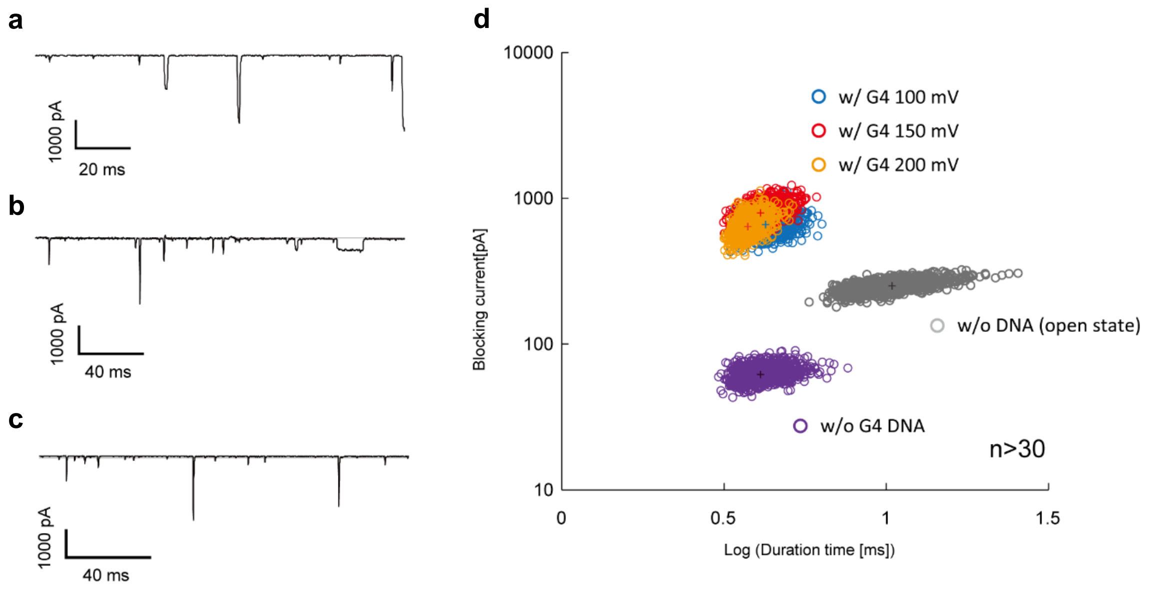

Fig. S19 Detection of DNA with G4 hybrid hold structure. (a-c) Current and time traces of G4 (2 μM) detection using SV28 nanopore (>6.4 nm in diameter) under different voltage applications: (a) 100 mV, (b) 150 mV, and (c) 200 mV. (d) The scatter plots of DNA with G4 under three different voltages, without G4 structure under 100 mV, and the pore open state of SV28 under 100 mV.

4. MD simulation of SVG28 nanopore (**Fig. S20**)

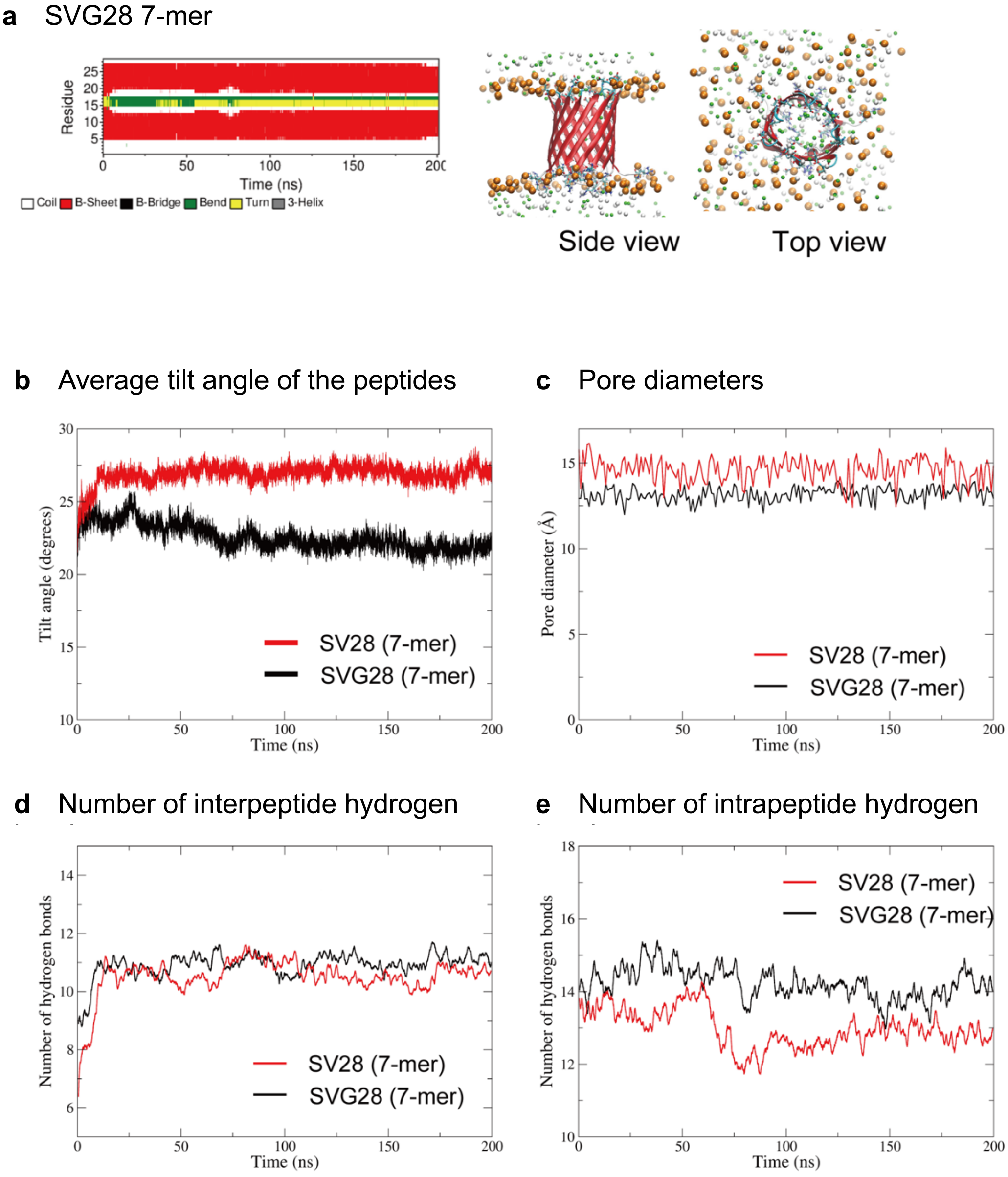

Fig. S20 MD simulation of SVG28. (a) The secondary structure and the top and side view of the SVG28. (b) Comparison of tilt angle of peptide pores between the SV28 and the SVG28. (c) Pore diameter of the SV28 and the SVG28 with 7-mers. (d) The number of interpeptide hydrogen bonding of SV28 and the SVG28. (e) The number of intrapeptide hydrogen bonding of SV28 and the SVG28.

5. Detection of poly-L-lysine using SVG28 nanopore (**Fig. S21 and S22**)

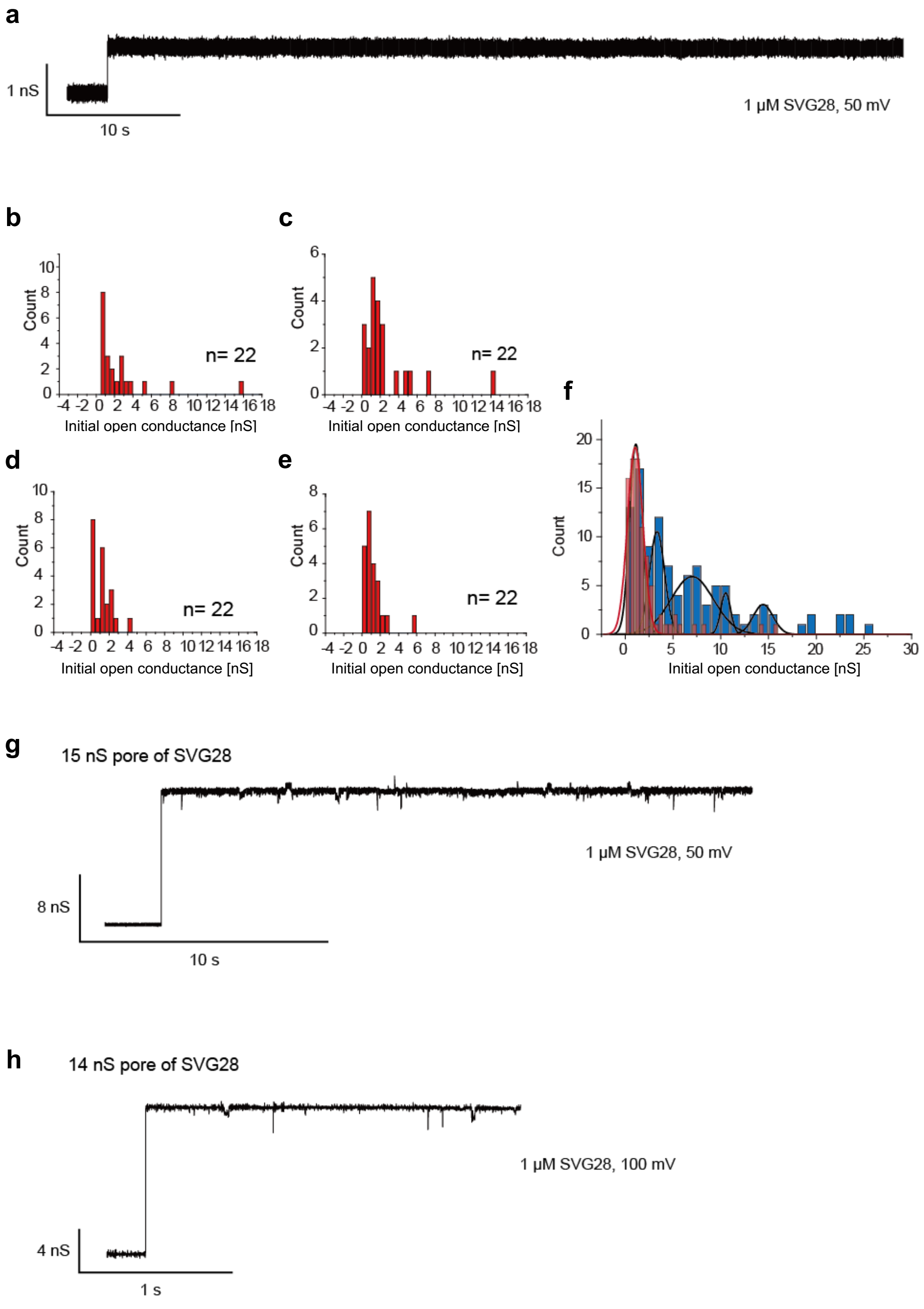

Fig. S21 Channel current recording of the SVG28 nanopore. (a) Typical current and time traces of the SVG28 pore under applying 50 mV. (b-e) The histograms of the initial pore-open-current of SVG28 form the baseline level (0 A) at (b) 50, (c) 100, (d) 150, and (e) 200 mV. f) Comparison of the conductance histograms between SV28 (blue) and SVG28 (red). (g, h) Typical current and time traces of two anormal large pore-formation of SVG28.

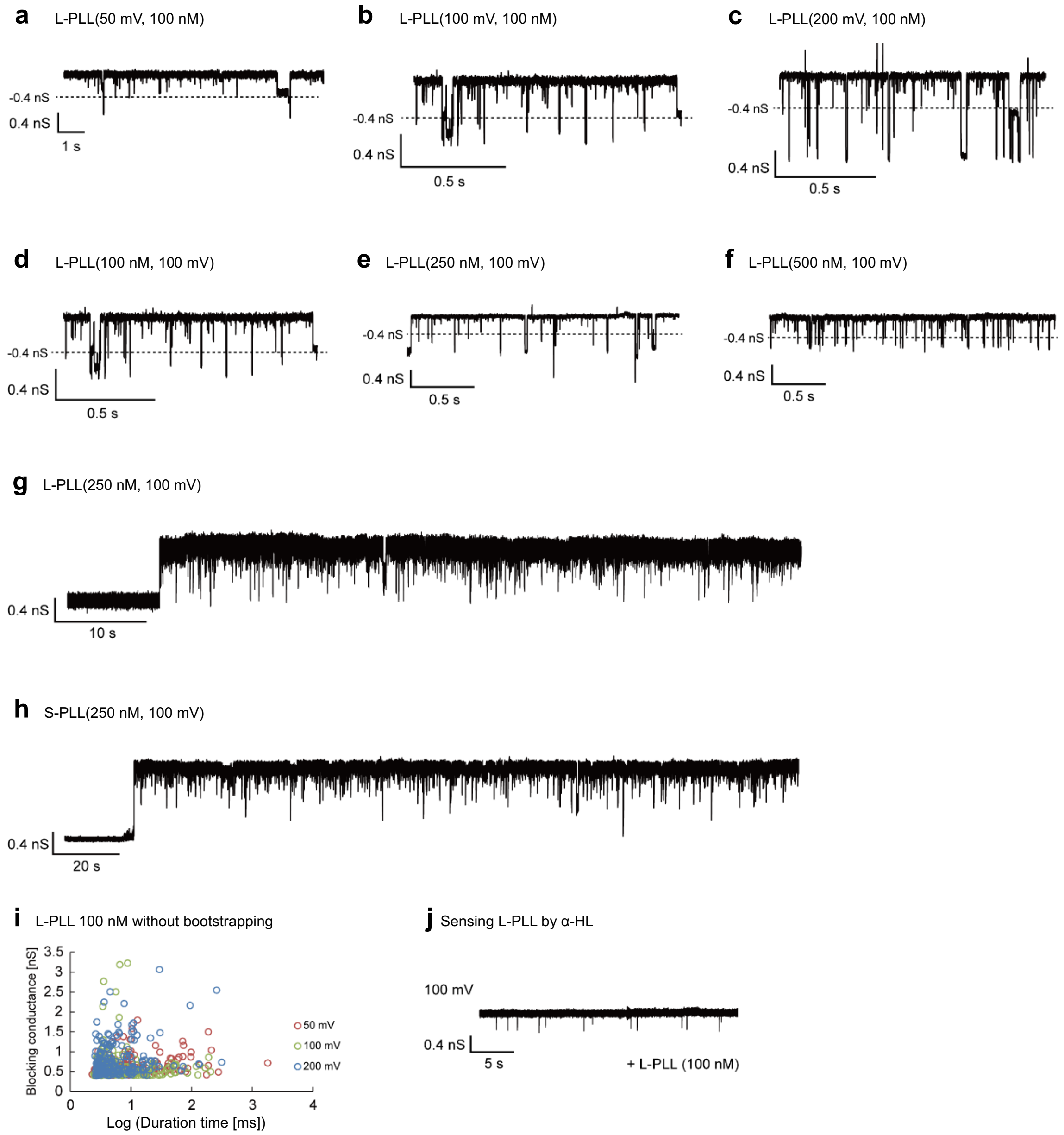

Fig. S22 Detection of poly-L-lysine (L (Longer) -PLL and S (Shorter) -PLL) using SVG28 nanopore (a-c) Typical current and time traces of L-PLL detection using SVG28 nanopore under different voltage applications: (a) 50 mV, (b) 100 mV, and (c) 200 mV. (d-f) Typical current and time traces of L-PLL detection using SVG28 nanopore with different concentration of the L-PLL: (d) 100 nM, (e) 250 nM, and (f) 500 nM. (g) The long detection experiment of the L-PLL using a single and initial pore-open state of the SVG28 nanopore. (h) The long detection experiment of the S-PLL using a single and initial pore-open state of the SVG28 nanopore. (i) The scatter plots of the L-PLL under three different voltages: 50 mV, 100 mV, and 200 mV. (j) Typical current and time trace of L-PLL detection using αHL nanopore.

6. References.

1 Van der Spoel, D. *et al.* GROMACS: fast, flexible, and free. *Journal of Computational Chemistry* **26**, 1701-1718, doi:10.1002/jcc.20291 (2005).

2 Humphrey, W., Dalke, A. & Schulten, K. VMD: visual molecular dynamics. *Journal of Molecular Graphics & Modelling* **14**, 33-38, doi:10.1016/0263-7855(96)00018-5 (1996).

3 Smart, O. S., Neduvelil, J. G., Wang, X., Wallace, B. A. & Sansom, M. S. P. HOLE: a program for the analysis of the pore dimensions of ion channel structural models. *Journal of Molecular Graphics & Modelling* **14**, 354-360, doi:10.1016/s0263-7855(97)00009-x (1996).

4 Paquet, A. Introduction of 9-fluorenylmethyloxycarbonyl, trichloroethoxycarbonyl, and benzyloxycarbonyl amine protecting groups into O-unprotected hydroxyamino acids using succinimidyl carbonates. *Canadian Journal of Chemistry* **60**, 976-980, doi:10.1139/v82-146 (1982).

5 Chang, C. D. *et al.* Preparation and properties of Nalpha-9-fluorenylmethyloxycarbonylamino acids bearing tert-butyl side-chain protection. *International Journal of Peptide and Protein Research* **15**, 59-66, doi:10.1111/j.1399-3011.1980.tb02550.x. (1980).

6 Taniguchi, A. *et al.* 'O-Acyl isopeptide method' for peptide synthesis: Solvent effects in the synthesis of Abeta1-42 isopeptide using 'O-acyl isodipeptide unit'. *Journal of Peptide Science* **13**, 868-874, doi:10.1002/psc.905 (2007).

7 Coin, I., Schmieder, P., Bienert, M. & Beyermann, M. The depsipeptide technique applied to peptide segment condensation: scope and limitations. *Journal of Peptide Science* **14**, 299-306, doi:10.1002/psc.928 (2008).

8 Yoshiya, T., Kawashima, H., Sohma, Y., Kimura, T. & Kiso, Y. O-acyl isopeptide method: efficient synthesis of isopeptide segment and application to racemization-free segment condensation. *Organic & Biomolecular Chemistry* **7**, 2894-2904, doi:10.1039/b903624e (2009).

9 Gullion, T. & Schaefer, J. Rotational-echo double-resonance NMR. *Journal of Magnetic Resonance* **81**, 196-200, doi:10.1016/0022-2364(89)90280-1 (1989).

10 Kawano, R. *et al.* Automated parallel recordings of topologically identified single ion channels. *Sci. Rep.* **3**, 1995, doi:10.1038/srep01995 (2013).

11 Kawano, R. *et al.* A portable lipid bilayer system for environmental sensing with a transmembrane protein. *PLOS ONE* **9**, doi:10.1371/journal.pone.0102427 (2014).

12 Ohara, M., Sekiya, Y. & Kawano, R. Hairpin DNA unzipping analysis using a biological nanopore array. *Electrochemistry* **84**, 338-341, doi:10.5796/electrochemistry.84.338 (2016).

13 Zakharian, E. & Reusch, R. N. Outer membrane protein A of Escherichia coli forms temperature-sensitive channels in planar lipid bilayers. *FEBS Lett.* **555**, 229-235, doi:10.1016/s0014-5793(03)01236-5 (2003).

14 Basle, A., Iyer, R. & Delcour, A. H. Subconductance states in OmpF gating. *Biochimica et Biophysica Acta* **1664**, 100-107, doi:10.1016/j.bbamem.2004.04.009 (2004).

15 Fahie, M. A. & Chen, M. Electrostatic interactions between OmpG nanopore and analyte protein surface can distinguish between glycosylated isoforms. *Journal of Physical Chemistry B* **119**, 10198-10206, doi:10.1021/acs.jpcb.5b06435 (2015).

16 Benz, R. Permeation of hydrophilic solutes through mitochondrial outer membranes: review on mitochondrial porins. *Biochimica et Biophysica Acta* **1197**, 167-196, doi:10.1016/0304-4157(94)90004-3 (1994).

17 Wolfe, A. J., Mohammad, M. M., Thakur, A. K. & Movileanu, L. Global redesign of a native beta-barrel scaffold. *Biochimica et Biophysica Acta* **1858**, 19-29, doi:10.1016/j.bbamem.2015.10.006 (2016).

18 Brogden, K. A. Antimicrobial peptides: pore formers or metabolic inhibitors in bacteria? *Nature Reviews Microbiology* **3**, 238-250, doi:10.1038/nrmicro1098 (2005).

19 Sekiya, Y., Sakashita, S., Shimizu, K., Usui, K. & Kawano, R. Channel current analysis estimates the pore-formation and the penetration of transmembrane peptides. *Analyst* **143**, 3540-3543, doi:10.1039/c8an00243f (2018).

20 Mani, R. *et al.* Membrane-dependent oligomeric structure and pore formation of beta-hairpin antimicrobial peptide in lipid bilayers from solid-state NMR. *Proceedings of the National Academy of Sciences of the United States of America* **103**, 16242-16247, doi:10.1073/pnas.0605079103 (2006).

21 Hille, B. *Ion channels of excitable membranes. 3rd edn*. (Sinauer, 2001).

22 Sharma, R. K., Agrawal, I., Dai, L., Doyle, P. S. & Garaj, S. Complex DNA knots detected with a nanopore sensor. *Nat. Commun.* **10**, doi:10.1038/s41467-019-12358-4 (2019).
